# Genome-wide profiling of DNA methylome and transcriptome in peripheral blood monocytes for major depression: A Monozygotic Discordant Twin Study

**DOI:** 10.1038/s41398-019-0550-2

**Published:** 2019-09-02

**Authors:** Yun Zhu, Eric Strachan, Emily Fowler, Tamara Bacus, Peter Roy-Byrne, Jinying Zhao

**Affiliations:** 1 Department of Epidemiology, College of Public Health and Health Professions and College of Medicine, University of Florida, Gainesville, FL USA; 2 Department of Psychiatry and Behavioral Sciences, University of Washington, Seattle, WA USA; 3 Department of Pediatrics, University of Washington, Seattle, WA USA

**Keywords:** Predictive markers, Comparative genomics

## Abstract

DNA methylation plays an important role in major depressive disorder (MDD), but the specific genes and genomic regions associated with MDD remain largely unknown. Here we conducted genome-wide profiling of DNA methylation (Infinium MethylationEPIC BeadChip) and gene expression (RNA-seq) in peripheral blood monocytes from 79 monozygotic twin pairs (mean age 38.2 ± 15.6 years) discordant on lifetime history of MDD to identify differentially methylated regions (DMRs) and differentially expressed genes (DEGs) associated with MDD, followed by replication in brain tissue samples. Integrative DNA methylome and transcriptome analysis and network analysis was performed to identify potential functional epigenetic determinants for MDD. We identified 39 DMRs and 30 DEGs associated with lifetime history of MDD. Some genes were replicated in postmortem brain tissue. Integrative DNA methylome and transcriptome analysis revealed both negative and positive correlations between DNA methylation and gene expression, but the correlation pattern varies greatly by genomic locations. Network analysis revealed distinct gene modules enriched in signaling pathways related to stress responses, neuron apoptosis, insulin receptor signaling, mTOR signaling, and nerve growth factor receptor signaling, suggesting potential functional relevance to MDD. These results demonstrated that altered DNA methylation and gene expression in peripheral blood monocytes are associated with MDD. Our results highlight the utility of using peripheral blood epigenetic markers and demonstrate that a monozygotic discordant co-twin control design can aid in the discovery of novel genes associated with MDD. If validated, the newly identified genes may serve as novel biomarkers or druggable targets for MDD and related disorders.

## Background

Major depressive disorder (MDD) affects over 350 million people worldwide and is projected to be the leading cause of disease burden by 2030^1^. Although the etiology of MDD involves genetic and environmental factors^2^, the precise underlying mechanisms remain poorly understood. A growing body of evidence has implicated a role for DNA methylation in MDD^3,4^, but the specific genes and biological pathways underlying MDD still remain unclear.

DNA methylation is influenced by genetic^5^, early life environment, and behavioral factors^6^, and is tissue and cell-type specific^7^. Thus, establishing the relationship between DNA methylation and mental illnesses, such as MDD requires the control of these potential confounding variables. Monozygotic (MZ) discordant twin pairs provide a powerful tool to examine the role of epigenetic mechanisms in depression, because they are matched on genotype, age, and sex. Moreover, identical twins reared together share early life familial environment^8^, which may contribute to the risk of depression later in life. At the time of this writing, several epigenome-wide association studies (EWAS) have been conducted to identify differentially methylated (DM) genes/regions associated with MDD using either unrelated individuals^4^ or twin pairs^9–12^. However, almost all previous studies utilized heterogeneous cell types from blood, buccal, or postmortem brain tissue samples. As different cell types have different DNA methylation profiles^7^, the use of homogeneous cell types such as purified monocytes should minimize confounding by cellular heterogeneity in epigenetic research. Moreover, because monocytes are key innate immune cells involved in inflammation, a mechanism known to be implicated in MDD^13^, identifying methylation changes in circulating monocytes is likely to provide mechanistic insight into disease pathogenesis. Further, the potential functional consequences of epigenetically altered genes on gene expression have not been adequately evaluated in previous studies.

Here we report findings from an integrated genome-wide profiling of DNA methylome and transcriptome in purified blood monocytes from 79 MZ twin pairs discordant for lifetime history of MDD, followed by replication in brain tissue and network analysis. Our goal is to identify key candidate genes and novel pathways associated with MDD.

## Methods

### Twin pairs

The current analysis included 79 MZ twin pairs discordant on lifetime history of MDD. Twins included in the current analysis were enrolled through the Mood and Methylation Study (MMS), an observational study designed to understand epigenetic and other molecular mechanisms underlying MDD using a co-twin control design. Detailed information regarding the study design, twin recruitment, clinical examination, and sample collection for the MMS has been described elsewhere^14^. Briefly, all twins enrolled in the MMS were members of the Washington State Twin Registry (WSTR), a community-based twin registry consisting of over 10,000 twin pairs^15^. Detailed methods for the construction of the registry and enrollment of twin pairs have been described previously^15^. All twin participants provided informed consent. Zygosity of the twin pairs included in the present study was confirmed using the 59 polymorphic SNPs included in the HumanMethylationEPIC array. Power analysis showed that we should have 80% power to detect a 1.1-fold change (FC) in DNA methylation at a genome-wide significance level^16^.

### MDD diagnosis

To identify twin pairs for the present study, we sent an introductory letter to WSTR members via postal mail and email that asked general questions about MDD. These letters were only sent to twins where one or both members were in Western Washington because of the need for an in-person study visit in Seattle. Interested twins were then interviewed by trained research staff using approved scripts to screen for likelihood of eligibility. After pre-screening, lifetime and current MDD diagnoses were determined using the Structured Clinical Interview for DSM-IV Research Version (SCID-4-RV). Interviews were administered via phone by a clinical psychologist (E.D.S.), who was blind to pre-screening clinical information about both the interviewee and his or her co-twin, and final diagnosis was confirmed by consulting the senior psychiatrist (P.P.R.B.). The final sample was drawn from a total of 693 clinical interviews and all eligible and interested MZ pairs who could complete the in-person visit prior to the end of the enrollment period were enrolled. A discordant pair was defined as a twin pair in which one twin met the criteria for lifetime history of MDD, and his/her co-twin did not.

### Inclusion/exclusion criteria

Only complete twin pairs were eligible for the present study. As over 85% of the twins in the WSTR are Caucasian (which reflects Washington State generally), all twin pairs included in the current analysis are Caucasian. Inclusion criteria included: (1) monozygosity determined by DNA analysis; (2) pairwise discordance on lifetime history of MDD; (3) aged 18 or older; (4) reared together; (5) Beck Depression Inventory II (BDI-II)^17^ score < 13 for the non-depressed twin (0–13 is the range for ‘minimal symptoms’ recommended by the BDI-II authors); and (6) willingness to provide blood samples. The primary exclusionary conditions included schizophrenia or other psychotic disorder, bipolar disorder, current substance use disorder, cancer within the past 5 years, autoimmune disorders, uncontrolled endocrine disorders, and uncontrolled sleep apnea.

### Other measures

Each twin was also asked to complete standard questionnaires regarding sociodemographic factors, lifestyle, early life experience, use of psychiatric medications, and disease history. Severity of current depressive symptoms was assessed by the BDI-II and the Quick Inventory of Depressive Symptomatology (QIDS-SR-16) at the time of the blood draw^18^. Self-reported early life stress was measured using the Adverse Childhood Experience (ACE)^19^ and Early Trauma Inventory (ETI)^20^ questionnaires. PTSD was not exclusionary in this study due to high comorbidity with MDD, but was diagnosed and recorded during the SCID-RV interviews. Participants reported their use of medications commonly prescribed for MDD along with the approximate duration of use (ranging from 2 weeks to >10 years). They were also asked to list any additional psychiatric medications they had taken. For the current analyses, medications were first categorized into types (i.e., antidepressants, benzodiazepines, mood-stabilizers, and other (i.e. Ritalin)), and then translated into a “yes/no” variable for each of these categories for each participant. A participant was defined as having a history of antidepressant use if he/she used any of the medications.

### Monocyte isolation and DNA/RNA extraction

Monocytes were isolated from fresh peripheral blood (collected into EDTA vacutainer tubes) using the Monocyte Isolation Kit II from Miltenyi Biotec (Auburn, CA, USA). DNA/RNA was isolated from monocytes using the AllPrep DNA/RNA/miRNA Universal Kit (Qiagen, CA, USA) according to manufacturer’s instructions. Double strand DNA and total RNA was quantified using PicoGreen Fluorometry. RNA integrity was assessed by capillary electrophoresis (e.g., Agilent BioAnalyzer 2100). Except for three samples with RNA integrity number (RIN) < 8 (one sample 7.0, one sample 7.6 and one sample 7.9), all other samples have RIN > 8. Of these, 88% samples have RIN > 9 (Table S1).

### DNA methylation profiling

The Infinium HumanMethylationEPIC BeadChip (Illumina Inc., CA, USA)^21^ was used for DNA methylation profiling. Genomic DNA (500 ng) was bisulfite converted using the EZ-96 DNA methylation kit (Zymo Research, CA). Modified DNA was then amplified, fragmented, and hybridized, followed by fluorescent staining and scanning on a HiScan scanner (Illumina Inc.). The methylation level of each probe was represented as a *β*-value ranging from 0 (unmethylated) to 1 (fully methylated). To ensure accuracy of the methylation assay, each batch included two CEPH DNA samples (Coriell Institute, NJ) as positive controls. Twin pairs were hybridized on the same chip to minimize batch effect.

### Methylation data pre-processing and QC

We first removed the following: (1) probes with a detection *p* > 0.05 in more than 20% of the samples; (2) probes with raw signal intensities greater or less than three SD from the mean; and (3) probes located on sex chromosomes (both X- and Y-chromosomes). Probes passing initial QC were then annotated to the UCSC (GRCh37/hg19), and those mapped to multiple locations or overlapping with known SNPs were further removed. The final analyses included 813,382 autosomal probes. All samples passed QC procedures. Prior to analysis, DNA methylation data were normalized with functional normalization using the R package *minfi*^22^. This method corrects for bias of different probe types and batch effects via an unsupervised approach^22^.

### Transcriptome profiling by RNA-seq

Monocyte gene expression was quantified by paired end RNA-seq (50 PE). Using sequence-specific Ribozero capture probes, total RNA (500 ng) was globin-depleted and rRNA-depleted, fragmented, and reverse transcribed. Trueseq libraries were quantified using PicoGreen Fluorimetry and sequenced on HiSeq 2500. Sequencing reads were aligned to the UCSC (GRCh37/hg19) by bowtie2^23^. Gene expression level measured as transcription per million reads (TPM) was quantified by RSEM v 1.12^24^. Genes with expression level below an appreciable level (log_2_(TPM + 1) < 1) in more than 5% samples were discarded. A total of 10,329 genes in all samples was included in the statistical analysis. At least 20 million reads per sample were captured in RNA-seq analysis (Table S1).

### Replication in the brain

To replicate the putative DMRs identified in blood monocytes, we downloaded brain DNA methylation data (HumanMethylation450K BeadChip) through the publicly available GEO database (GSE41826). The brain tissue was collected by the NICHD Brain Bank of Developmental Disorders^25^, and DNA methylation data were generated in sorted neuronal nuclei from 58 postmortem brain tissue, including 29 MDD patients and 29 controls (51.7% female, mean age 32.6 ± 16.0 years old). Demographic information of the brain donors was described previously^25^.

To replicate the putative DEGs identified in blood monocytes, we downloaded brain gene expression data (RNA-seq) through publicly available GEO database (GSE101521). The expression data were generated in 59 postmortem brain tissue (29% females, mean age 49.3 ± 20.3 years old) collected by The Division of Molecular Imaging and Neuropathology at the New York State Psychiatric Institute and Columbia University. In brief, the whole-exome gene expression was examined in non-psychiatric controls (*N* = 29), MDD suicides (*N* = 21), and MDD non-suicides (*N* = 9) in postmortem brain tissue (dorsal lateral prefrontal cortex, Brodmann Area 9). All participants were free of medications. Detailed information of the brain samples was described previously^26^. Depression was diagnosed by the structured clinical interview (DSM-IV) in both datasets.

### Statistical analysis

The primary goal of our statistical analysis is to identify differentially methylated genes/regions (DMRs) associated with MDD. The overall study design and analytical plan was shown in Fig. S1.

### Identifying differentially methylated regions (DMRs) associated with MDD

As DNA methylation between adjacent probes could be functionally and/or spatially correlated, identifying genomic regions containing biologically relevant probes should be preferable compared to single CpG analysis. To achieve this, we employed a mixed-model designed specifically for discordant twin pairs^27^

$${\mathrm {Log}}_2\left[ {\frac{{{\mathrm {Methylation}}\,{\mathrm {level}}\,{\mathrm {in}}\,{\mathrm {depressed}}\,{\mathrm {twin}}}}{{{\mathrm {Methylation}}\,{\mathrm {level}}\,{\mathrm {in}}\,{\mathrm {nondepressed}}\,{\mathrm {cotwin}}}}} \right] = \alpha + {\sum} {\beta _ix_i} + {\sum} {1|y_j}$$

In this model, *β*_*i*_ represents regression coefficient for the *i*th fixed-effect variable (e.g., age, sex), and *y*_*j*_ denotes the random-effect of the *j*th covariate (e.g. batch effect). The intercept *α* stands for the mean FC in DNA methylation between depressed and non-depressed twins within a pair. By testing the null hypothesis of *α* = 0, we were able to test whether DNA methylation alteration (*α* > 0—hypermethylation, *α* < 0—hypomethylation) at a specific CpG site was associated with MDD or not. The effect of environmental factors on DNA methylation was assessed by testing, where *β* equals 0 or not (*β* > 0 indicates that the corresponding covariable causes hypermethylation, *β* < 0 indicates that it causes hypomethylation at the CpG site being tested in the depressed twins). This model allows for establishing a link between environmental exposure, epigenetic alteration, and depression. In this analysis, we adjusted for covariates including twin age, sex, BMI, smoking (pack-year), alcohol consumption, family income, and education level as fixed effect, and batch was included as random effect.

Region-based analysis was performed using results from single CpG analysis (*p*-value and effect size) by the program DMRcate^28^, which identifies genomic regions harboring CpG sites and accounts for correlations between adjacent probes. Here we defined a DMR as a region containing ≥ 5 correlated probes (peak probe *p* < 0.01 and correlation among probes ≥ 0.30), and a significant DMR was defined as a region with *q* < 0.05 after correcting for total number of 6858 regions. Putative DMRs were ranked and annotated to genomic features based on the UCSC database (GRCh37/hg19). Genomic features of DMRs were compared to the null distribution of CpG probes included in the MethylationEPIC array^21^.

### Differentially expressed genes (DEGs) associated with MDD

Using the statistical model described above, we identified DEGs associated with lifetime history of MDD, adjusting for same covariates.

### Integrated methylome and transcriptome analysis

To examine the impact of DNA methylation on gene expression in peripheral blood monocytes, we calculated partial correlation coefficients (corrected for twin age, sex, BMI, smoking, alcohol, family income, and education) between DNA methylation and *cis*-acting gene expression for each probe in all samples. Here *cis-*acting was defined as correlation between DNA methylation of a putative gene with its own expression (±5 kb to a tested probe). We used a conservative threshold to determine significant negative (partial correlation < −0.84) or positive (partial correlation > 0.84) correlation. This threshold was obtained by randomly permuting DNA methylation and gene expression datasets for all twins. The cutoff was based on the fifth percentile of the empirical distribution of partial correlation coefficients, assuming no correlation between methylation and gene expression across all participants (null hypothesis). To further examine the role of DNA methylation in gene regulation, we used the Fisher’s Exact test implemented in the GeneOverlap software^29^ to determine the statistical significance of genes showing both differential methylation and differential expression in relation to MDD status.

### Methods used for the replication in brain

For each of the 39 DMRs identified in blood monocytes, we tested the association of each probe within each region with depression (y/n) using logistic regression, adjusting for age and sex. Similar analysis was conducted to test the association of each putative DEG with depression, adjusting for age, sex, RIN, and brain PH. Multiple testing corrected for 39 DMRs or 30 DEGs using false discovery rate (FDR).

### Co-methylation and co-expression networks

To examine the correlation patterns among putative DMRs and identify genes that are co-methylated, we performed network analyses using the weighted gene correlation network analysis (WGCNA). Co-methylation or co-expression networks were constructed separately in depressed twins and their non-depressed co-twins using all genes. Differential methylation or expression networks were identified by comparing the connectivity of differential genes between the two groups. This analysis included 322 DMRs showing nominal association (raw *p* < 0.001) with MDD. Similar analysis was used to identify co-expression networks (326 genes with raw *p* < 0.001 was included). Network visualization was done using CytoScape^30^.

### Functional-enrichment analysis

To explore the potential functional relevance of the identified DM or differentially expressed (DE) genes, we conducted functional enrichment analysis using the program DEPICT^31^. We first tested whether the putative genes (*p* < 0.001) are enriched in GWAS loci for major depression. A total of 746 independent SNPs previously associated with major depression (*p* < 1 × 10^−5^) was downloaded from the GWAS catalog^32^ for this analysis. We then tested whether the identified genes are enriched in gene sets related to antidepressants by extracting 283 drug target genes from the Open Targets database^33^.

### Sensitivity analysis

To examine whether ACE modulates the association between DNA methylation and MDD, we further adjusted for ACE (y/n) in the above-described statistical model. Similarly, we tested the influence of antidepressants usage (y/n) or history of PTSD (y/n) on the relationship between DNA methylation and MDD.

### Control for multiple comparisons

In the above-described analyses, we adjusted for multiple testing by FDR and FDR-adjusted *p* (i.e., *q*-value) < 0.05 was considered statistically significant.

## Results

Table 1 shows the characteristics of the twins (mean age 38.2 ± 15.6 years, 68.4% females). Of the 79 twins with MDD, 8 twins have current MDD and 71 have MDD in the past. In addition, among the 79 depressed twins, 12 twins were under current medications, and 30 twins were taking medications in the past. Of note, 12 non-depressed co-twins were taking antidepressants prescribed for a number of different clinical indications outside of MDD, including chronic pain, anxiety, insomnia, and post-partum mood symptoms. None of the non-depressed co-twins with a history of antidepressant use met criteria for any MDD at any point in their lives. Except for current BDI-II score, PTSD, and use of antidepressants, depressed twins did not differ significantly from their non-depressed co-twins.

**Table 1:** Clinical characteristics of twin pairs participating in the MMS

Variable	Non-depressed co-twin (*N* = 79)	Depressed twin (*N* = 79)	*p*-value^a^
Age, mean (SD), years	38.2 (15.6)	38.2 (15.6)	–
Female, no. (%)	54 (68.4)	54 (68.4)	–
Body mass index, mean (SD) (kg/m^2^)	26.9 (6.5)	27.0 (6.9)	0.82
Smoking, mean (SD) (pack/year)	2.3 (9.4)	2.4 (8.3)	0.83
AUDIT-C score, mean (SD)^b^	3.6 (2.6)	3.4 (2.8)	0.70
Education below high school, no. (%)	6 (7.6)	4 (5.1)	0.43
Family income less than $20,000, no. (%)	6 (7.6)	7 (8.9)	0.53
BDI-II score, mean (SD)	3.1 (5.1)	6.6 (6.5)	0.003
Exposure to ACE, no. (%)	8 (10.1)	9 (11.4)	0.66
History of PTSD, no. (%)	1 (1.3)	6 (7.6)	0.014
Use of antidepressants, no. (%)	12 (15.2)	42 (53.2)	0.006

*BDI-II* Beck Depressive Inventory II, *ACE* adverse childhood experiences, *PTSD* post-traumatic stress disorder

^a^*p*-values were calculated using paired *t*-test

^b^Score based on alcohol use disorders identification test (AUDIT-C)

### DMRs associated with lifetime history of MDD

**Fig. 1:**
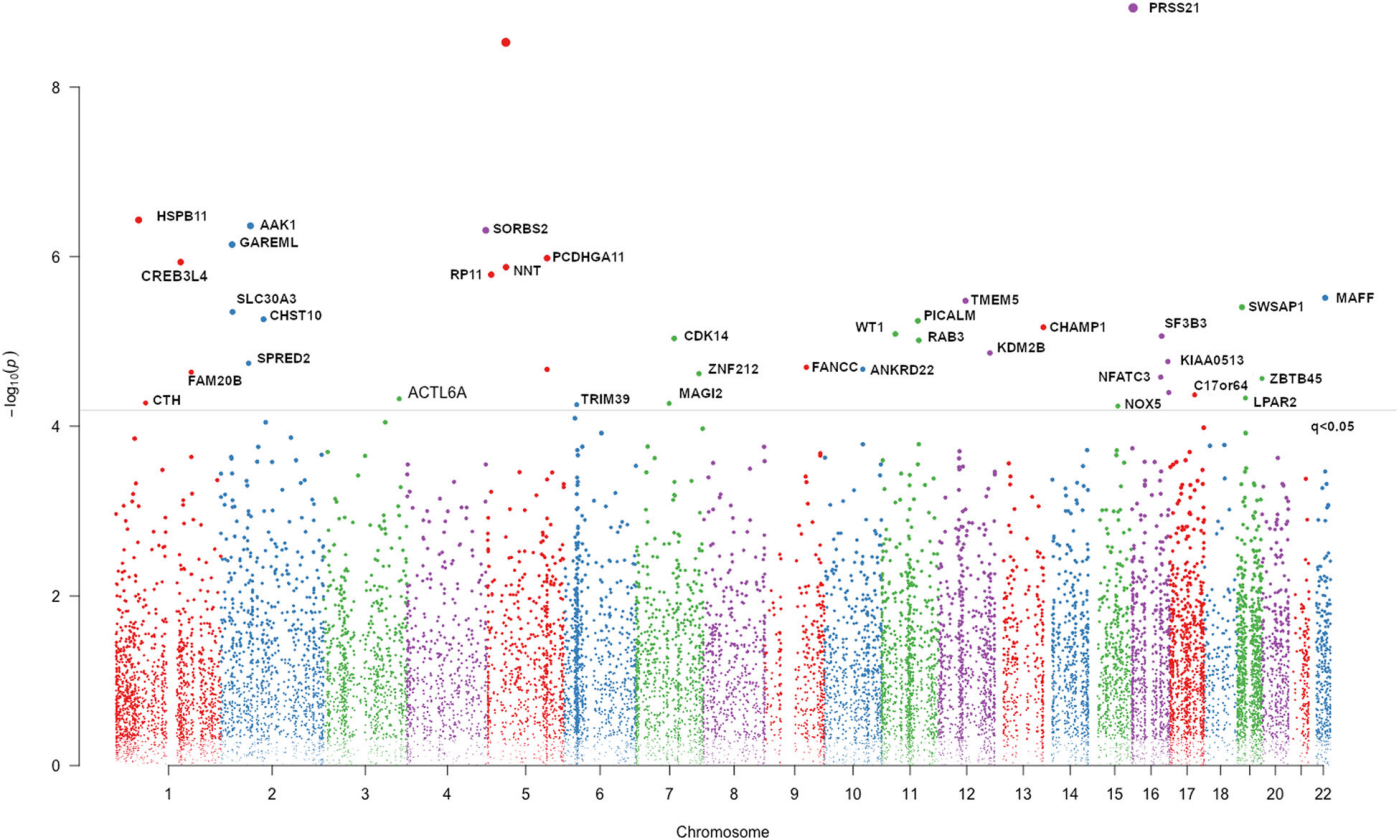
Manhattan plot displaying the DMRs associated with MDD in monozygotic discordant twin pairs (*N* = 79 pairs). The *p*-values (−log_10_) of each DMR are plotted against their respective positions on each chromosome. The genome-wide threshold (*q* < 0.05) is indicated with a red line

We identified 39 DMRs (annotated to 36 unique genes) significantly associated with MDD at *q* < 0.05 (Table 2). Of these, 33 DMRs are hyper-methylated, and 6 are hypo-methylated in relation to MDD. Figure 1 shows a Manhattan plot for these DMRs. The genomic distribution of these DMRs is shown in Fig. S2, which shows that the identified DMRs are enriched in the first exon and promoter regions but depleted in intergenic regions. In relation to CpG context, the identified DMRs are largely located within CpG Islands (CGIs).

**Table 2:** Significant DMRs associated with lifetime history of MDD in MZ discordant twin pairs

Chr	Start (bp)	End (bp)	Size (bp)	Nearest gene	# of probes	Peak *P*^a^	Region *P*^b^	Mean FC^c^	Mean difference (%)^d^	Peak difference (%)^e^
16	2,866,834	2,868,001	1168	*PRSS21*	10	6.28 × 10^−4^	1.16 × 10^−9^	1.13	4.06	14.28
5	43,037,123	43,037,666	544	*ANXA2R*	7	2.99 × 10^−4^	2.96 × 10^−9^	1.15	0.46	1.26
1	54,411,017	54,412,009	993	*HSPB11*	18	1.90 × 10^−3^	3.69 × 10^−7^	1.05	0.72	4.02
2	69,870,526	69,871,424	899	*AAK1*	8	5.70 × 10^−4^	4.32 × 10^−7^	1.05	2.48	8.45
4	186,732,926	186,733,331	406	*SORBS2*	8	3.53 × 10^−3^	4.89 × 10^−7^	1.14	6.98	13.36
2	26,395,359	26,395,859	501	*GAREML*	8	5.55 × 10^−3^	7.20 × 10^−7^	1.08	0.29	0.81
5	140,800,398	140,800,983	586	*PCDHGA11*	10	1.85 × 10^−3^	1.04 × 10^−6^	1.11	8.02	11.54
1	153,940,616	153,941,285	670	*CREB3L4*	6	2.76 × 10^−3^	1.16 × 10^−6^	1.12	11.12	15.53
5	43,602,380	43,603,353	974	*NNT*	17	2.76 × 10^−3^	1.33 × 10^−6^	1.06	1.03	6.84
5	8,457,538	8,458,392	855	*RP11*	9	4.84 × 10^−3^	1.63 × 10^−6^	0.89	−3.24	−8.65
22	38,598,577	38,599,166	590	*MAFF*	9	5.79 × 10^−3^	3.06 × 10^−6^	1.05	4.46	7.30
12	64,173,610	64,174,367	758	*TMEM5*	9	6.05 × 10^−3^	3.32 × 10^−6^	1.04	1.73	4.48
19	11,484,448	11,485,452	1005	*SWSAP1*	14	8.08 × 10^−5^	3.94 × 10^−6^	1.05	0.56	1.61
2	27,485,922	27,486,460	539	*SLC30A3*	8	3.43 × 10^−3^	4.49 × 10^−6^	1.07	1.52	6.90
2	101,034,246	101,034,295	50	*CHST10*	6	5.93 × 10^−3^	5.48 × 10^−6^	1.10	7.51	8.30
11	85,779,252	85,780,378	1127	*PICALM*	10	1.85 × 10^−3^	5.72 × 10^−6^	1.06	5.28	13.31
13	114,814,024	114,814,401	378	*CHAMP1*	5	7.42 × 10^−3^	6.81 × 10^−6^	1.09	7.08	13.05
11	32,454,216	32,455,025	810	*WT1*	8	2.18 × 10^−3^	8.16 × 10^−6^	1.06	2.34	6.02
16	70,557,411	70,557,707	297	*SF3B3*	10	7.70 × 10^−3^	8.65 × 10^−6^	1.06	3.32	8.43
7	90,224,158	90,225,380	1223	*CDK14*	11	9.78 × 10^−4^	9.23 × 10^−6^	0.99	−1.06	−3.14
11	87,908,134	87,908,805	672	*RAB38*	7	6.00 × 10^−3^	9.72 × 10^−6^	1.09	1.26	5.74
12	122,019,031	122,019,117	87	*KDM2B*	5	8.20 × 10^−3^	1.37 × 10^−5^	0.91	−8.86	−11.79
16	85,096,632	85,097,151	520	*KIAA0513*	5	6.05 × 10^−3^	1.73 × 10^−5^	0.94	−3.88	−7.55
2	65,594,021	65,595,186	1166	*SPRED2*	6	9.76 × 10^−5^	1.81 × 10^−5^	1.09	2.29	5.55
9	98,079,646	98,080,622	977	*FANCC*	10	2.64 × 10^−4^	2.02 × 10^−5^	1.07	6.64	11.76
10	90,611,604	90,612,228	625	*ANKRD22*	7	2.33 × 10^−3^	2.13 × 10^−5^	1.07	5.22	8.13
5	140,777,344	140,777,655	312	*PCDHA1*	9	4.87 × 10^−3^	2.14 × 10^−5^	1.11	8.87	13.05
1	178,994,834	178,995,133	300	*FAM20B*	8	2.76 × 10^−3^	2.31 × 10^−5^	1.06	5.27	9.33
7	148,936,572	148,937,410	839	*ZNF212*	9	3.03 × 10^−3^	2.40 × 10^−5^	1.06	1.46	4.08
16	68,118,822	68,119,261	440	*NFATC3*	9	7.94 × 10^−3^	2.64 × 10^−5^	1.05	4.97	8.83
19	59,030,662	59,031,081	420	*ZBTB45*	7	6.62 × 10^−3^	3.75 × 10^−5^	1.02	0.97	2.52
16	87,351,006	87,351,824	819	*C16orf95*	10	1.63 × 10^−3^	4.00 × 10^−5^	1.05	5.03	13.48
17	58,499,300	58,500,186	887	*C17orf64*	9	9.07 × 10^−4^	4.27 × 10^−5^	0.93	−5.43	−9.35
19	19,739,060	19,739,414	355	*LPAR2*	8	6.05 × 10^−3^	4.65 × 10^−5^	1.04	1.81	4.80
3	179,280,056	179,280,746	691	*ACTL6A*	9	3.97 × 10^−3^	4.75 × 10^−5^	1.02	1.48	4.75
1	70,876,598	70,877,381	784	*CTH*	9	5.78 × 10^−3^	5.33 × 10^−5^	1.06	1.23	3.90
7	78,400,383	78,400,769	387	*MAGI2*	5	6.61 × 10^−3^	5.39 × 10^−5^	1.09	8.22	12.42
6	30,297,174	30,297,941	768	*TRIM39*	10	2.76 × 10^−3^	5.57 × 10^−5^	1.09	8.93	14.69
15	69,222,400	69,223,018	619	*NOX5*	7	6.22 × 10^−3^	5.79 × 10^−5^	0.88	−4.11	−10.37

Chr: chromosome, Size: region size, # of probes: number of probes in the region

^a^Adjusted for twin age, sex, BMI, smoking, alcohol consumption, education, and family income

^b^Adjusted for a total number of 6858 regions

^c^Mean fold change (FC) in DNA methylation level across all CpG probes in a region. FC > 1 represents hypermethylated, whereas FC < 1 represents hypomethylated (depressed twin vs. non-depressed co-twin)

^d^Mean methylation difference across all probes in the region between depressed twins and their non-depressed co-twins

^e^Methylation difference of the peak probe in the region between depressed twins and their non-depressed co-twins

### DEGs associated with MDD

We identified 30 DEGs (14 upregulated, 16 downregulated) associated with lifetime history of MDD at *q* < 0.05 (Table 3). A Manhattan plot of these putative DEGs is shown in Fig. S3.

**Table 3:** Significant DEGs associated with lifetime history of MDD in MZ discordant twin pairs

Chr	Start (bp)	End (bp)	Size (bp)	Nearest gene	FC^a^	*P* ^b^	*q* ^c^
2	215,996,329	216,082,955	86,626	*PECR*	1.49	7.00 × 10^−8^	7.10 × 10^−3^
1	185,292,384	185,294,372	1988	*AL356273.3*	1.32	5.12 × 10^−7^	2.59 × 10^−2^
1	44,800,225	44,805,990	5765	*PLK3*	2.48	9.35 × 10^−7^	2.89 × 10^−2^
5	71,197,646	71,208,130	10,484	*GUSBP9*	1.65	1.29 × 10^−6^	2.89 × 10^−2^
8	63,015,079	63,039,171	24,092	*GGH*	1.27	1.78 × 10^−6^	2.89 × 10^−2^
6	11,538,278	11,583,524	45,246	*TMEM170B*	8.02	2.47 × 10^−6^	2.89 × 10^−2^
11	64,223,799	64,226,254	2455	*TRPT1*	0.75	2.90 × 10^−6^	2.89 × 10^−2^
16	19,701,934	19,718,235	16,301	*KNOP1*	0.78	3.36 × 10^−6^	2.89 × 10^−2^
16	3,222,325	3,236,221	13,896	*ZNF200*	0.82	3.39 × 10^−6^	2.89 × 10^−2^
6	30,617,709	30,626,395	8686	*MRPS18B*	0.57	3.48 × 10^−6^	2.89 × 10^−2^
7	66,682,164	66,811,464	129,300	*RABGEF1*	1.46	3.65 × 10^−6^	2.89 × 10^−2^
22	49,900,229	49,918,458	18,229	*ALG12*	1.19	3.71 × 10^−6^	2.89 × 10^−2^
12	52,076,841	52,082,084	5243	*AC025259.1*	0.69	5.78 × 10^−6^	3.62 × 10^−2^
19	19,668,796	19,683,509	14,713	*ZNF101*	0.61	6.16 × 10^−6^	3.62 × 10^−2^
1	151,156,629	151,159,749	3120	*TNFAIP8L2*	0.16	6.35 × 10^−6^	3.62 × 10^−2^
8	33,473,386	33,513,601	40,215	*TTI2*	0.80	6.79 × 10^−6^	3.62 × 10^−2^
13	41,457,559	41,470,882	13,323	*RGCC*	4.27	7.44 × 10^−6^	3.74 × 10^−2^
9	122,144,058	122,159,819	15,761	*NDUFA8*	0.59	7.73 × 10^−6^	3.74 × 10^−2^
11	93,741,591	93,764,749	23,158	*C11orf54*	0.59	8.66 × 10^−6^	3.99 × 10^−2^
16	85,690,084	85,751,129	61,045	*C16orf74*	1.62	9.17 × 10^−6^	4.04 × 10^−2^
7	80,742,538	80,922,359	179,821	*SEMA3C*	1.81	1.03 × 10^−5^	4.10 × 10^−2^
19	21,397,119	21,427,573	30,454	*ZNF493*	1.65	1.05 × 10^−5^	4.10 × 10^−2^
11	4,384,897	4,393,696	8799	*TRIM21*	0.28	1.18 × 10^−5^	4.30 × 10^−2^
19	52,949,379	52,962,911	13,532	*ZNF816*	0.72	1.21 × 10^−5^	4.30 × 10^−2^
10	43,436,841	43,483,179	46,338	*ZNF487*	1.44	1.23 × 10^−5^	4.30 × 10^−2^
19	57,466,663	57,477,570	10,907	*ZNF772*	0.84	1.27 × 10^−5^	4.30 × 10^−2^
22	42,509,968	42,519,802	9834	*RRP7A*	0.43	1.34 × 10^−5^	4.31 × 10^−2^
21	36,069,941	36,073,166	3225	*CBR1*	0.35	1.36 × 10^−5^	4.31 × 10^−2^
2	98,619,106	98,731,126	112,020	*MGAT4A*	2.80	1.60 × 10^−5^	4.91 × 10^−2^
1	145,911,350	145,918,837	7487	*PEX11B*	0.67	1.66 × 10^−5^	4.94 × 10^−2^

Chr: Chromosome, Size: length of gene in base pair

^a^Fold change (FC) in gene expression level between depressed twins and their non-depressed co-twins

^b^Adjusted for twin age, sex, BMI, smoking, alcohol consumption, education, and family income

^c^Adjusted for a total number of 10,329 genes

### Replication in the brain

Of the 39 DMRs identified in blood, 10 regions contain at least one CpG probe showing significant association (same direction) with MDD (*q* < 0.05) in the brain after adjustments for covariates and total number of probes in each region (Table S2).

Of the 30 DEGs identified in blood monocytes, two genes (*NDUFA8, GUSBP9*) were also significantly associated with MDD (*q* < 0.05) in the brain (Table S3). While the expression level of the *NDUFA8* gene was lower in MDD patients than that in controls (i.e., downregulated) in both blood and brain, the *GUSBP9* gene was in an opposite direction (i.e., upregulated in blood, but downregulated in the brain between cases and controls).

### Genome-wide integration of DNA methylome and transcriptome in blood monocytes

**Fig. 2:**
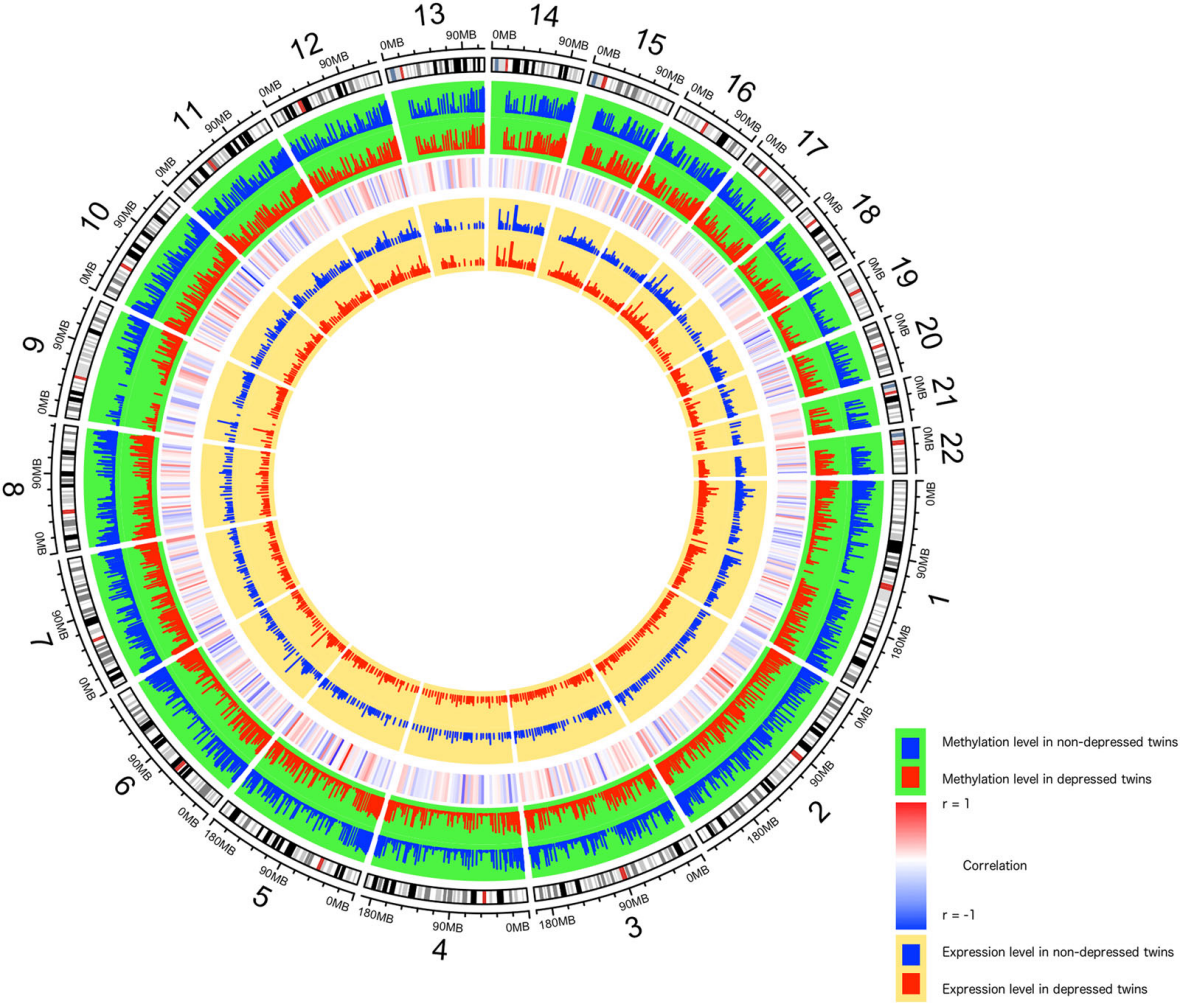
Circos plot showing the genome-wide relationship between DNA methylation and gene expression in peripheral blood monocytes in relation to major depression. The outermost ring displays chromosome numbers and bands. The second ring (green) shows differential methylation in depressed (red) and non-depressed (blue) twins. The third ring shows Pearson correlation between DNA methylation and gene expression in 500 bp bin in the combined samples. The inner most circle (yellow) represents mRNA differential expression in depressed (red) and non-depressed (blue) twins. The height of the histogram bins indicates the level of DNA methylation or gene expression

Figure 2 displays the genome-wide correlation patterns between DNA methylation and *cis*-acting gene expression in peripheral blood monocytes. It shows that DNA methylation was largely negatively (74% of the correlation pairs) correlated with gene expression, but positive correlations were also observed. Interestingly, we found that the correlation patterns between DNA methylation and gene expression vary by genomic locations, with genes located about 1 kb upstream (negative) or downstream (positive) of the transcription start site (TSS) showing stronger correlation, whereas those located in between showing weak or no correlation (Fig. S4). These correlation pairs (Table S4) involve 140 distinct methylated loci (from 64 unique genes) and 60 corresponding expression regions (representing 57 unique genes). The observed correlation between DNA methylation and gene expression appears to be independent of MDD, because after additional adjustment for MDD status (y/n) in the correlation analysis, majority (93.8%) of the correlation pairs remain statistically significant.

### Differential network analysis

Our co-methylation network analysis identified three differential co-methylation modules containing at least 50 genes. Table S5 lists the co-methylation modules along with hub genes (genes with highest module membership) and biological pathways in each module. A total of 304 genes (94.4% of the total 322 genes showing nominal association with MDD) were assigned to at least one module. The largest module (Fig. S5) comprises 167 genes involved in four biological processes. The network connectivity (measured by node degrees) of two pathways in this module significantly differs between depressed and non-depressed twins. For example, the node connectivity for a pathway named “negative regulation of neuron apoptotic process” in depressed twins was significantly higher than that in non-depressed twins (3.8 vs. 2.6, *P* = 7.15 × 10^−5^), whereas the node connectivity of another pathway called “stress-activated protein kinase signaling cascade” was significantly lower in depressed twins compared to their non-depressed co-twins (2.8 vs. 4.2, *P* = 2.21 × 10^−5^).

Our co-expression network analysis identified five modules containing 292 genes (Table S6). The largest module comprises 94 genes involved in eight biological processes. Of these, the “positive regulation of cytokine secretion” pathway showed significant difference between depressed and non-depressed twins (Fig. S6)

### Functional-enrichment analysis

The identified DM genes are significantly enriched in pathways related to stress-activated protein kinase signaling cascade, neuron apoptotic process, negative regulation of insulin receptor signaling, mTOR signaling, and nerve growth factor receptor signaling pathways (Table S7). The differentially expressed genes are enriched in biological processes related to “positive regulation of cytokine secretion” and “regulation of response to stress”. Gene overlapping analysis revealed that the putative DM genes are 2.44 times more likely to be differentially expressed (*P* = 1.1 × 10^−4^), and are significantly overrepresented in previous GWAS loci for major depression (2.32 times, *P* = 2.4 × 10^−4^). Moreover, these DM genes are significantly enriched in drug targets related to antidepressants (2.83 times, *P* = 7.6 × 10^−5^), and are highly expressed in tissues/cell types related to the nervous, endocrine, and urogenital systems (Fig. S7). Together, these results suggest a potential functional role of altered DNA methylation in MDD pathogenesis.

### Sensitivity analysis

Additionally adjustment for childhood traumatic experience (ACE) slightly attenuated the association between DNA methylation and MDD, but results remained largely unchanged. After further correction for use of antidepressants or PTSD, the association of the *MAFF* gene with MDD disappeared, but other genes remained statistically significant. Results for sensitivity analysis are shown in Table S8. It appears that additionally adjustments for adverse childhood events (ACE), PTSD, or use of antidepressants did not have an appreciable effect on the association between gene expression and MDD (Table S9).

## Discussion

Using a MZ discordant co-twin control design, we identified 39 DMRs (33 hypermethylated, 6 hypomethylated) and 30 differentially expressed genes (14 upregulated, 16 downregulated) associated with lifetime history of MDD, after accounting for clinical covariates and multiple testing. These DM or expressed genes are significantly enriched in biological processes related to neuronal function, stress response, insulin regulation, mTOR signaling, and cytokine secretion, suggesting potential relevance to MDD pathogenesis. Moreover, the identified DMR genes are overrepresented in GWAS loci and drug targets related to antidepressants. Integrated DNA methylome and transcriptome analysis revealed that DNA methylation was both negatively and positively correlated with gene expression in peripheral blood monocytes. To the best of our knowledge, this is the first integrated DNA methylome and transcriptome analysis in purified blood monocytes for lifetime history of MDD using a relatively large number of MZ discordant pairs from a community-based population cohort.

In a recent meta-analysis examining the association between blood DNA methylation and depressive symptoms among middle-aged and elderly persons^4^, DNA methylation at three CpG sites were significantly associated with depressive symptoms. One of these probes (cg12325605) could be replicated in our study at a nominal level (*P* = 2.28 × 10^-4^). Multiple prior studies have also reported possible associations of DNA methylation in stress-related genes^34^ or genome-wide associations using unrelated individuals or twins in various tissues (peripheral or postmortem brain)^10,35,36^. However, due to the heterogeneity of MDD phenotypes and many other differences between studies (e.g., racial/ethnic groups, age ranges of participants, tissue/cell types, risk factor profiles, etc.), it is challenging to compare results across different scenarios, and no conclusive genes have been identified so far. Of the limited number of existing EWAS on MDD, the present study represents the first to utilize purified blood monocytes in a relatively large sample of MZ twin pairs discordant on MDD.

Of the 36 annotated DMR genes, the *PRSS21* gene showed the most significant association with MDD. The methylation level of this gene in depressed twins is on average 1.13-fold as high as that in their non-depressed co-twins. This gene (also known as testisin) encodes a glycosylphosphatidylinositol (GPI)-linked serine protease, which is a member of the trypsin family of serine proteases. A growing body of evidence demonstrates that the brain can co-opt the activities of these serine proteases and their receptors/inhibitors to regulate various processes including synaptic activity, learning, and social behavior^37^. Aberrant activity of these molecules may contribute to neurological disorders such as Alzheimer’s disease, Parkinson’s disease, traumatic brain injury, and stroke^37^. Another significantly hypermethylated gene is *HSPB11*, which encodes a family member of the heat shock proteins (HSPs) that are produced in response to stressful conditions^38^. Although the exact mechanisms behind the association of *HSPB11* hypermethylation with MDD are unknown, HSPs are involved in protein misfolding and aggregation, which have been implicated in neurodegenerative and neuropsychiatric disorders^39^. Other top-ranked DMR genes, such as *AAK1, SORBS2*, and *GAREM2*, are also abundantly expressed in the brain and may affect MDD susceptibility through a variety of biological processes. For example, the *AAK1* gene is involved in intracellular vesicle trafficking, a mechanism that is essential for neurotransmitter release and recycling of synaptic vesicle proteins^40^. Inhibition of *AAK1* activity may provide a novel therapeutic target for treating neuropsychiatric and neurodegenerative disorders^41^. The *SORBS2* gene encodes the Arg protein tyrosine kinase-binding protein 2 (ArgBP2), downregulation of which was previously associated with mood disorders^42^. The *GAREM2* gene encodes an adapter protein that regulates MAPK/ERK signaling, which is involved in neuronal plasticity and resilience in psychiatric disorders^43^. Further, the identified DMR genes are enriched in neuron apoptosis^44^, nerve growth factor^45^, stress-activated protein kinase signaling^46^, insulin receptor regulation^47^, and mTOR signaling^48^, suggesting potential relevance to MDD pathogenesis.

Of the differentially expressed genes associated with MDD, the peroxisomal *trans*-2-enoyl-CoA reductase (*PECR*) gene showed the strongest association. This gene is involved in mitochondrial energy production by catalyzing the reduction of enoyl-CoAs to acyl-CoAs, and genetic polymorphisms in this gene were associated with alcohol dependence^49^. The differentially expressed genes are enriched in regulation of cytokine secretion and oxidative stress responses, lending further support for the critical roles of inflammation and oxidative stress in major depression^13^.

Previous studies have demonstrated that zinc plays an essential role in synaptic activity and neuronal plasticity, and that zinc deficiency was associated with behavioral impairments^50^, neurodegenerative disorders, and mood disorders including depression^51^. In line with these findings, we found that several zinc family genes are DM (e.g., *SLC30A3, ZNF212, ZBTB45, SWSAP1, WT1, TRIM39*) or differentially expressed (e.g., *ZNF200, ZNF101, ZNF493, ZNF816, ZNF487, ZNF772*) between depressed twins and their non-depressed co-twins. These findings provide further support for a potential important role of zinc dysregulation in depression, and suggest that DNA methylation may modulate the effect of zinc function on depression susceptibility. Together, our results may unravel novel molecular pathways underlying MDD pathogenesis.

Although DNA methylation is generally believed to cause gene silencing, a global analysis of the extent and pattern of DNA methylation with gene expression in human blood monocytes is still lacking. Here we demonstrated that monocyte DNA methylation can be both positively and negatively correlated with gene expression, although there appears to be a trend that the correlations are predominantly negative in promoter regions. These results are in agreement with previous studies reporting both positive and negative correlations between DNA methylation and gene expression in blood and brain^52^. Interestingly, we found that the relationship between DNA methylation and *cis*-acting gene expression appears to vary by genomic locations, with methylation of putative genes located upstream of TSS showing predominantly negative correlations, whereas those located downstream of TSS showing largely positive correlations with gene expression. While the negative correlation may result from the interference with transcription factor binding or recruiting repressors such as histone deacetylases^51^, the positive correlation between DNA methylation and gene expression may be attributed to the high level of DNA methylation in the gene body of highly transcribed genes. In addition, it has been shown that DNA methylation of a promoter or an enhancer can activate transcription of a target gene and therefore is positively correlated with gene expression^53^. Together, our integrated DNA methylome and transcriptome analysis confirmed the importance of DNA methylation in gene regulation and identified key candidate genes whose role in depression are modulated by epigenetic changes.

MDD is a highly heterogeneous disorder involving the joint or interactive effects of many genes in multiple pathways. Traditional methods that model the effect of a single gene cannot capture the complicated biological pathways implicated in MDD. Using a network-based approach, we identified co-methylated or co-expressed modules containing coordinated genes across different genomic loci. These findings support the hypothesis that altered DNA methylation is interdependent and that the blood monocytes methylome and transcriptome comprise a complex network of interacting processes.

Our study has several limitations. First, in spite of using 79 MZ discordant twin pairs, our study is still underpowered and thus we might have missed important disease-related genes, especially those with small individual effect size. As such, the current analysis focused on region-based rather than single probe analysis. Our results should be considered as a proof of concept rather than conclusive. Second, although we used purified blood monocytes for DNA methylation and gene expression profiling, our sample still includes other cell types, such as macrophages or dendritic cells, each of which may have different epigenetic profiles. However, our sample has nearly 97% purity and we believe confounding by cellular heterogeneity should not be a major concern for our study. Moreover, given the cell-type-specific nature of DNA methylation, it is unclear to what extent our results derived from peripheral blood could reflect methylation changes in the brain. However, monocytes are one of the key components of the innate immunity system, dysfunction of which has been implicated in neuropsychiatric disorders including depression^54^, and accumulating evidence indicated that epimutations may not be limited to the affected organ (e.g., brain) but could also be detected in peripheral blood^55^. Further, many of the identified DMR genes are abundantly expressed and some could be replicated in the brain. These putative genes detected in easily accessible tissues, such as blood are suitable for biomarkers. Third, the twin participants were evaluated for lifetime history of MDD and some non-depressed co-twins might ultimately develop an episode of MDD. The mean age of our sample was 38, which suggests that a large portion of the participants had already passed the peak risk period of young adulthood^56^. Fourth, due to the uneven distribution of the CpG probes on the Illumina methylation arrays, we cannot entirely exclude the possibility that the observed correlation patterns between monocytes DNA methylation and *cis*-acting gene expression is likely reflecting the way the Illumina methylation array was designed. Whether the observed correlation patterns between DNA methylation and gene expression is due to true biology or artifact could be verified when whole genome bisulfite sequencing data become available in the near future. Moreover, like all other observational studies, establishing causality between DNA methylation and MDD is impossible in the present study. Fifth, of the putative CpG probes identified in this study, there is a considerable likelihood that some CpG sites, especially those with smaller effect sizes, could be due to technical artifact but not reflect true biology. Other potential limitations include the limited genome coverage of the EPIC array used in our study, and the uncertainty to generalize our findings to other racial/ethnic groups.

Our study has several strengths. First, as MZ twin pairs share almost identical genotypes, age, sex, and early familial environment (e.g., in utero environment), as well as many unknown or unmeasured factors, the use of a MZ discordant co-twin control design minimizes or eliminates potential confounding by these factors. Second, we profiled DNA methylome and transcriptome in purified blood monocytes, which minimizes confounding by cellular heterogeneity. Moreover, we used the structured clinical interview (DSM-IV) for depression diagnosis.

In summary, our results demonstrated a critical role of altered DNA methylation and gene expression in MDD, and identified key candidate genes and pathways underlying MDD pathogenesis. If validated, the newly identified genes and pathways may serve as novel therapeutic targets for MDD and related disorders.

## Supplementary information

Supplementary Figure Legend.

Figure S1

Figure S2

Figure S3

Figure S4

Figure S5

Figure S6

Figure S7

Table S1

Table S2

Table S3

Table S4

Table S5

Table S6

Table S7

Table S8

Table S9
