# Supplementary material for: Genome-wide profiling of DNA methylome and transcriptome in peripheral blood monocytes for major depression: A Monozygotic Discordant Twin Study
Source: Transl Psychiatry. 2019 Sep 2;9:215. doi: 10.1038/s41398-019-0550-2 (PMC6718674; doi:10.1038/s41398-019-0550-2)
Supplement: Supplementary file 1 — Supplementary Figure Legend. [file 41398_2019_550_MOESM1_ESM.docx]

**Figure S1.** Flowchart of study design and analytical plan.

**Figure S2.** Genomic distribution of the identified DMRs. (a) Genomic distribution of identified DMRs associated with MDD. (b) CpG content of DMRs

**Figure S3.** Manhattan plot displaying the DEGs associated with MDD in monozygotic discordant twin pairs (N=79 pairs) The P-values (-log_10_) of each DEG are plotted against their respective positions on each chromosome. The genome-wide threshold (q<0.05) is indicated with a blue line.

**Figure S4.** Genome-wide partial correlation patterns between DNA methylation and *cis*-expression (±5kb). Mean level (green) and 95% confidence interval (orange) of the normalized correlation between DNA methylation and gene expression across multiple genes in the combined samples was plotted against genomic position in relation to transcription start site (TSS).

**Figure S5.** The largest co-methylation module associated with MDD in depressed twins in comparison to their non-depressed co-twins. The network connectivity (as measured by node degrees) for the negative regulation of neuron apoptotic process (green) in depressed twins is significantly higher compared to non-depressed co-twins (3.8 vs 2.6, p-value =7.15×10^-5^). In contrast, the network connectivity of the stress-activated protein kinase signaling cascade (purple) is significantly lower in depressed twins than that in non-depressed co-twins (2.8 vs 4.2, P=2.21×10^-5^).

**Figure S6.** The largest co-expression module for the identified DEGs. The network connectivity (as measured by node degrees) for the positive regulation of cytokine secretion (green) in depressed twins is significantly higher compared to non-depressed co-twins (3.8 vs 2.6, p-value =7.15×10^-5^).

**Figure S7.** Tissue/cell types enrichment of the identified DMRs. It shows that the MDD-related DMRs are significantly enriched in the nervous system, endocrine system, and urogenital system. P-values of the enrichment analysis adjusted for a total number of 209 tissue/cell types. The red line indicates q<0.05.
