# Supplementary material for: Genome-wide profiling of DNA methylome and transcriptome in peripheral blood monocytes for major depression: A Monozygotic Discordant Twin Study
Source: Transl Psychiatry. 2019 Sep 2;9:215. doi: 10.1038/s41398-019-0550-2 (PMC6718674; doi:10.1038/s41398-019-0550-2)
Supplement: Supplementary file 2 — Figure S1 [file 41398_2019_550_MOESM2_ESM.pptx]

## Slide 1
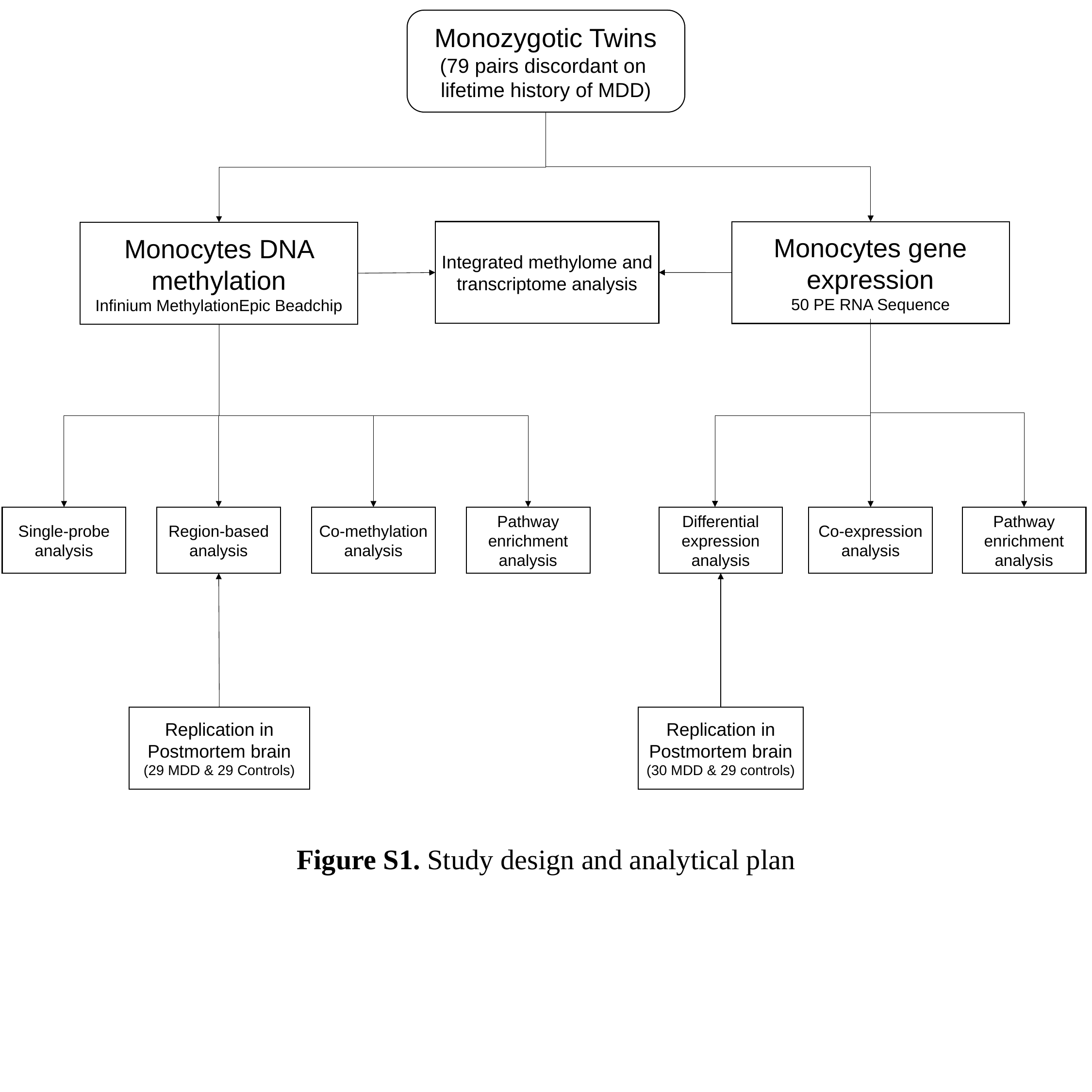

Monozygotic Twins
(79 pairs discordant on
lifetime history of MDD)
Integrated methylome and transcriptome analysis
Monocytes gene expression
50 PE RNA Sequence
Monocytes DNA methylation
Infinium MethylationEpic Beadchip
Single-probe analysis
Region-based analysis
Co-methylation analysis
Pathway enrichment analysis
Differential expression analysis
Co-expression analysis
Pathway enrichment analysis
Replication in Postmortem brain
(29 MDD & 29 Controls)
Replication in
Postmortem brain
(30 MDD & 29 controls)
Figure S1. Study design and analytical plan
