# Supplementary material for: Genome-wide profiling of DNA methylome and transcriptome in peripheral blood monocytes for major depression: A Monozygotic Discordant Twin Study
Source: Transl Psychiatry. 2019 Sep 2;9:215. doi: 10.1038/s41398-019-0550-2 (PMC6718674; doi:10.1038/s41398-019-0550-2)
Supplement: Supplementary file 3 — Figure S2 [file 41398_2019_550_MOESM3_ESM.docx]

(a) (b)

**Figure S2.** (a) Genomic distribution of identified DMRs associated with MDD. (b) CpG content of DMRs
