# Supplementary material for: Genome-wide profiling of DNA methylome and transcriptome in peripheral blood monocytes for major depression: A Monozygotic Discordant Twin Study
Source: Transl Psychiatry. 2019 Sep 2;9:215. doi: 10.1038/s41398-019-0550-2 (PMC6718674; doi:10.1038/s41398-019-0550-2)
Supplement: Supplementary file 4 — Figure S3 [file 41398_2019_550_MOESM4_ESM.pdf]

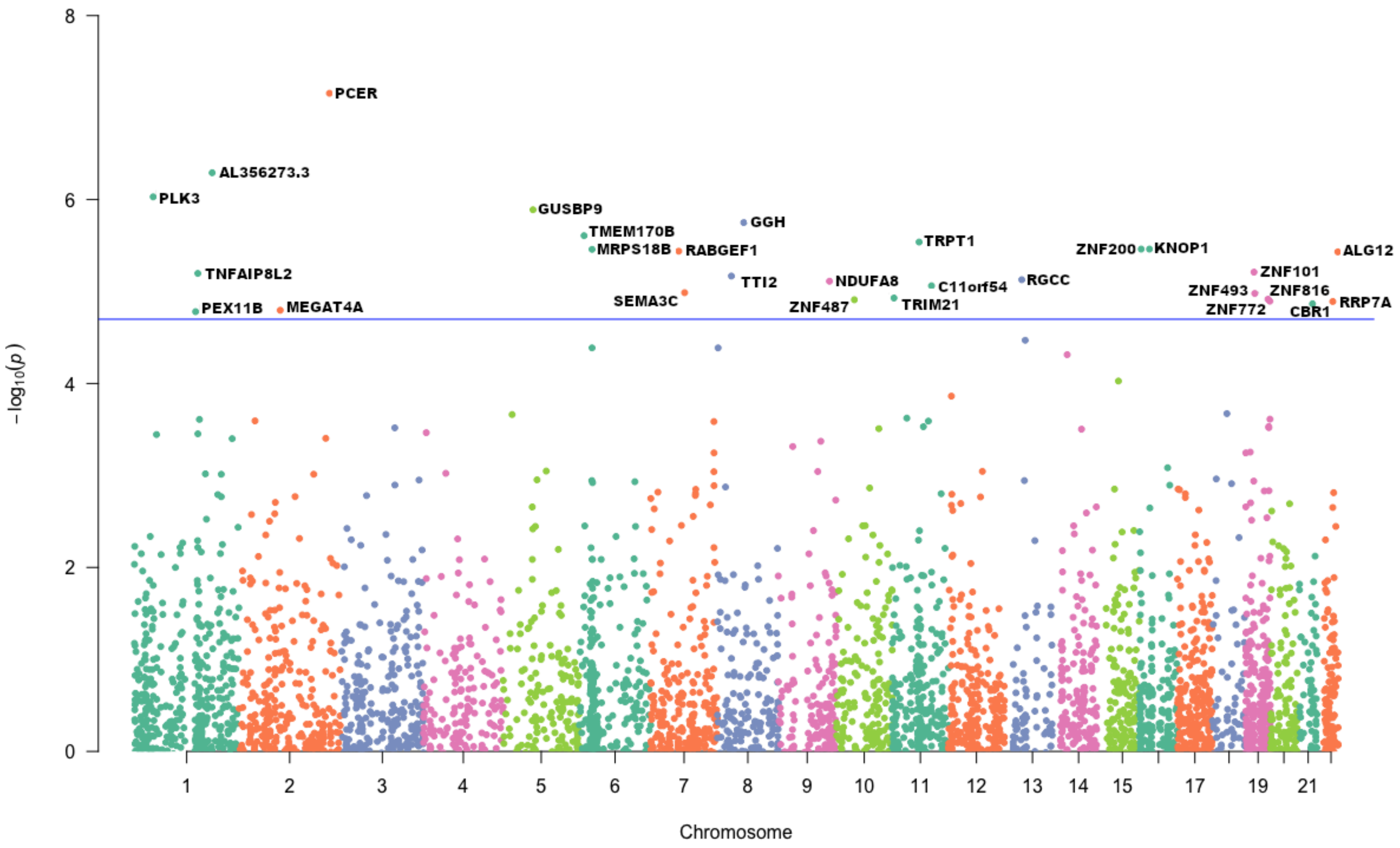

Figure S3. Manhattan plot displaying the DEGs associated with MDD in monozygotic discordant twin pairs (N=79 pairs). The P-values (-$\log_{10}$) of each DEG are plotted against their respective positions on each chromosome. The genome-wide threshold (q<0.05) is indicated with a blue line.
