# Supplementary material for: Genome-wide profiling of DNA methylome and transcriptome in peripheral blood monocytes for major depression: A Monozygotic Discordant Twin Study
Source: Transl Psychiatry. 2019 Sep 2;9:215. doi: 10.1038/s41398-019-0550-2 (PMC6718674; doi:10.1038/s41398-019-0550-2)
Supplement: Supplementary file 5 — Figure S4 [file 41398_2019_550_MOESM5_ESM.docx]

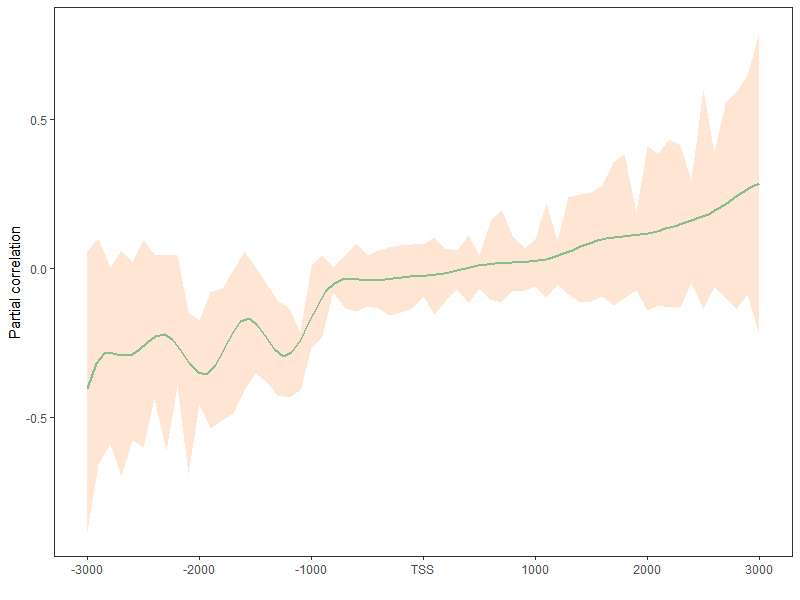


**Figure S4.** Genome-wide partial correlation patterns between DNA methylation and *cis*-expression (±5kb). Mean level (green) and 95% confidence interval (orange) of the normalized correlation between DNA methylation and gene expression across multiple genes in the combined samples was plotted against genomic position in relation to transcription start site (TSS).
