# Supplementary material for: Genome-wide profiling of DNA methylome and transcriptome in peripheral blood monocytes for major depression: A Monozygotic Discordant Twin Study
Source: Transl Psychiatry. 2019 Sep 2;9:215. doi: 10.1038/s41398-019-0550-2 (PMC6718674; doi:10.1038/s41398-019-0550-2)
Supplement: Supplementary file 6 — Figure S5 [file 41398_2019_550_MOESM6_ESM.docx]

**
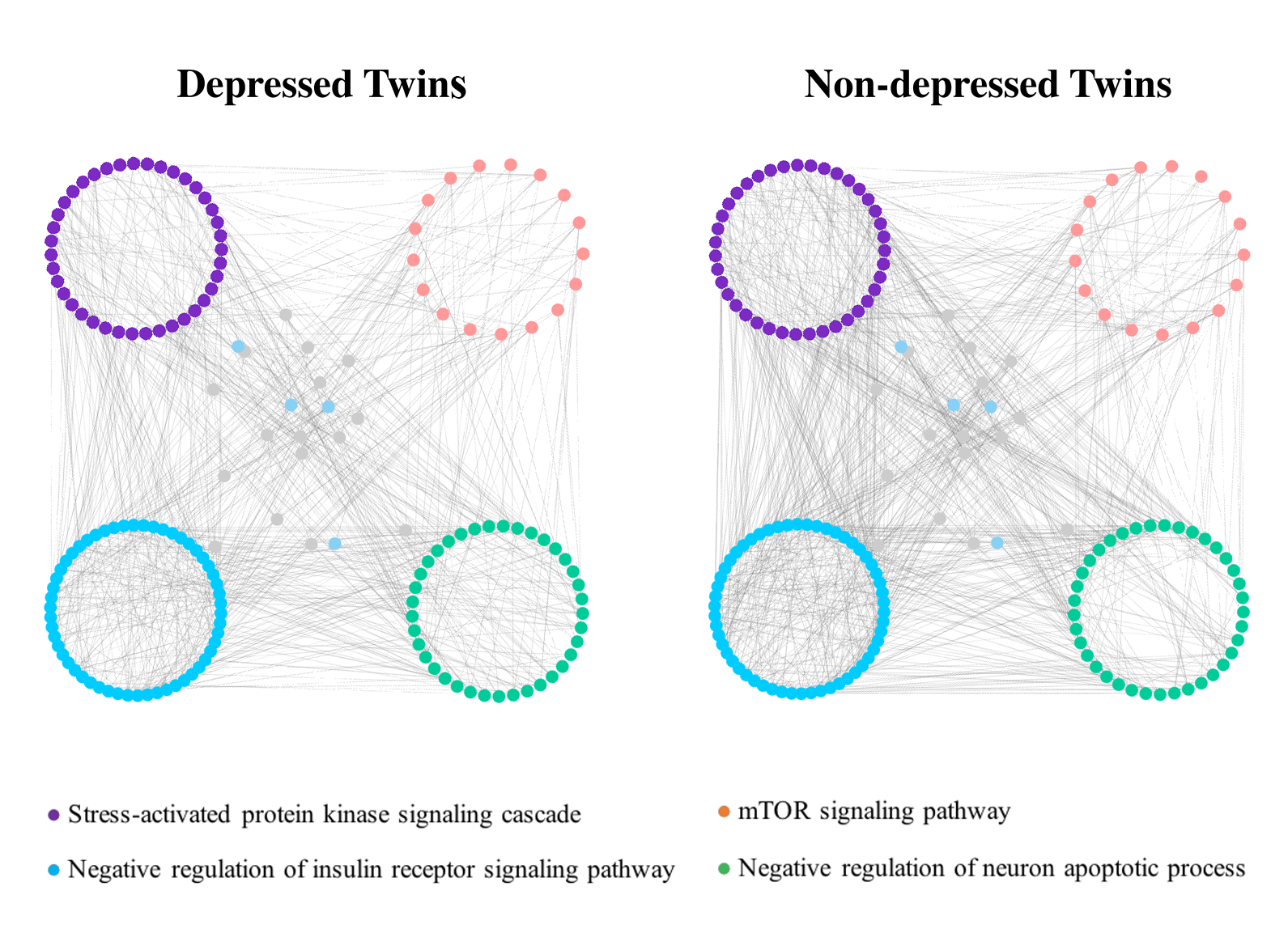
**

**Figure S5.** The largest co-methylation module associated with MDD in depressed twins in comparison to their non-depressed co-twins. The network connectivity (as measured by node degrees) for the negative regulation of neuron apoptotic process (green) in depressed twins is significantly higher compared to non-depressed co-twins (3.8 vs 2.6, p-value =7.15×10^-5^). In contrast, the network connectivity of the stress-activated protein kinase signaling cascade (purple) is significantly lower in depressed twins than that in non-depressed co-twins (2.8 vs 4.2, P=2.21×10^-5^).
