# Supplementary material for: Genome-wide profiling of DNA methylome and transcriptome in peripheral blood monocytes for major depression: A Monozygotic Discordant Twin Study
Source: Transl Psychiatry. 2019 Sep 2;9:215. doi: 10.1038/s41398-019-0550-2 (PMC6718674; doi:10.1038/s41398-019-0550-2)
Supplement: Supplementary file 7 — Figure S6 [file 41398_2019_550_MOESM7_ESM.docx]

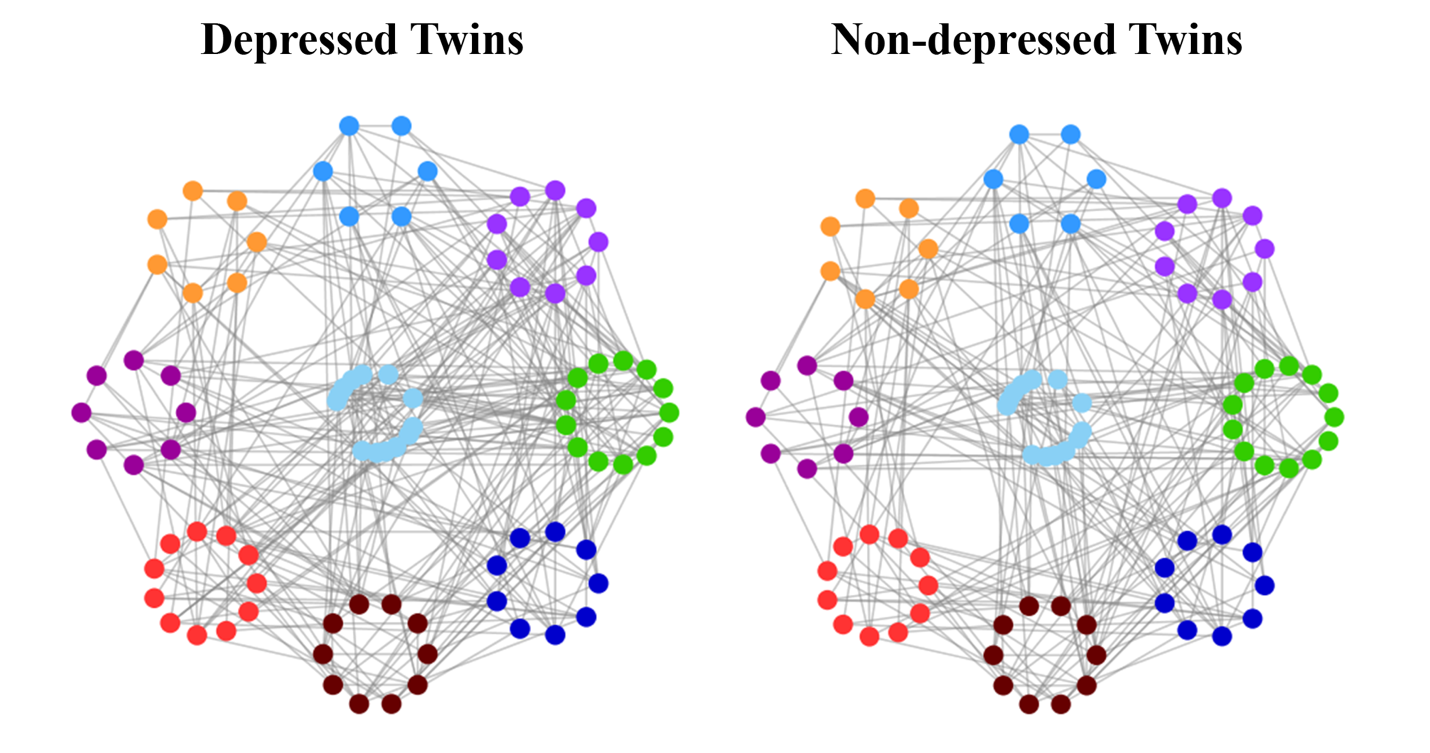


**Figure S6.** The largest co-expression module for the identified DEGs. The network connectivity (as measured by node degrees) for the positive regulation of cytokine secretion (green) in depressed twins is significantly higher compared to non-depressed co-twins (3.8 vs 2.6, p-value =7.15×10^-5^).
