# Supplementary material for: Genome-wide profiling of DNA methylome and transcriptome in peripheral blood monocytes for major depression: A Monozygotic Discordant Twin Study
Source: Transl Psychiatry. 2019 Sep 2;9:215. doi: 10.1038/s41398-019-0550-2 (PMC6718674; doi:10.1038/s41398-019-0550-2)
Supplement: Supplementary file 8 — Figure S7 [file 41398_2019_550_MOESM8_ESM.docx]

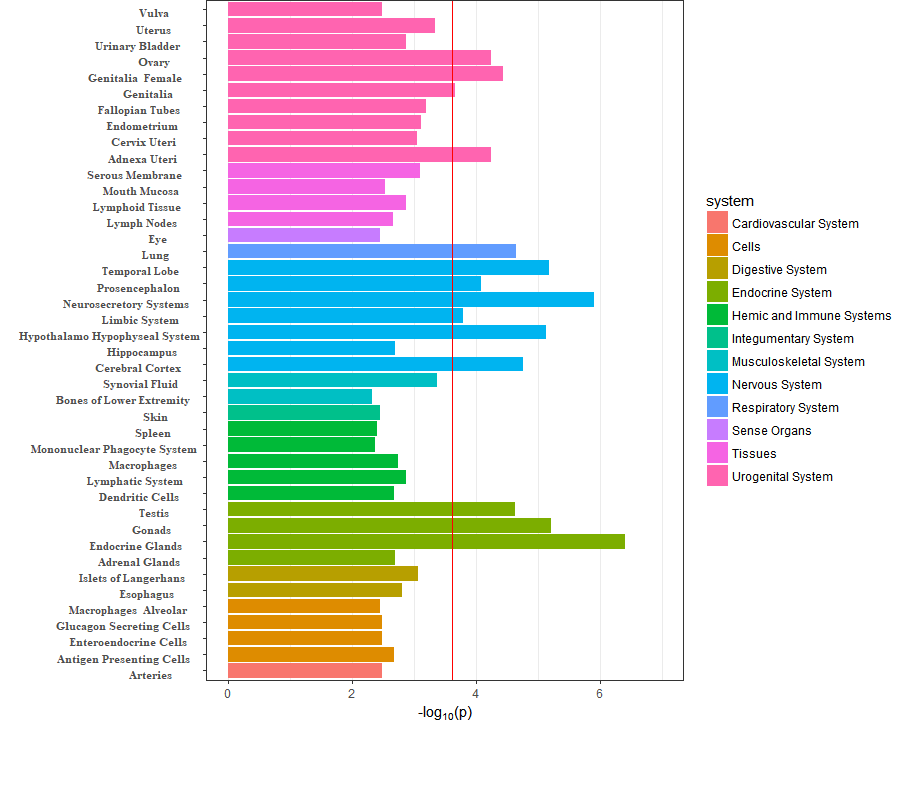
 **Figure S7.** Tissue/cell types enrichment of the identified DMRs. It shows that the MDD-related DMRs are significantly enriched in the nervous system, endocrine system, and urogenital system. P-values of the enrichment analysis adjusted for a total number of 209 tissue/cell types. The red line indicates q<0.05.
