# Supplementary material for: Genome-wide profiling of DNA methylome and transcriptome in peripheral blood monocytes for major depression: A Monozygotic Discordant Twin Study
Source: Transl Psychiatry. 2019 Sep 2;9:215. doi: 10.1038/s41398-019-0550-2 (PMC6718674; doi:10.1038/s41398-019-0550-2)
Supplement: Supplementary file 9 — Table S1 [file 41398_2019_550_MOESM9_ESM.docx]

**Table S1**. QC information for RNA-seq

| Study ID | RIN | Total Reads | Aligned Reads | % Aligned Reads | QC30 |
| --- | --- | --- | --- | --- | --- |
| 1 | 8.5 | 35,589,699 | 29,603,511 | 83.18% | 1 |
| 2 | 8 | 32,382,943 | 29,332,470 | 90.58% | 0.97 |
| 3 | 10 | 30,947,356 | 27,781,441 | 89.77% | 0.99 |
| 4 | 9.7 | 33,105,942 | 26,239,769 | 79.26% | 1 |
| 5 | 9.5 | 26,766,472 | 24,135,328 | 90.17% | 1 |
| 6 | 9.8 | 30,252,083 | 27,157,295 | 89.77% | 1 |
| 7 | 9.5 | 37,706,116 | 30,858,685 | 81.84% | 1 |
| 8 | 9.6 | 28,348,653 | 23,841,217 | 84.10% | 1 |
| 9 | 9.3 | 22,842,796 | 17,943,016 | 78.55% | 1 |
| 10 | 9.7 | 35,065,192 | 31,614,777 | 90.16% | 0.99 |
| 11 | 9.8 | 31,837,624 | 28,338,669 | 89.01% | 1 |
| 12 | 8.9 | 21,712,939 | 19,435,252 | 89.51% | 1 |
| 13 | 9 | 32,172,418 | 28,890,831 | 89.80% | 1 |
| 14 | 9.8 | 29,465,971 | 26,380,884 | 89.53% | 1 |
| 15 | 9.9 | 26,577,598 | 24,289,267 | 91.39% | 1 |
| 16 | 8.7 | 21,382,699 | 19,208,078 | 89.83% | 1 |
| 17 | 9.8 | 26,897,611 | 21,797,824 | 81.04% | 1 |
| 18 | 9.7 | 20,482,031 | 18,724,673 | 91.42% | 0.99 |
| 19 | 10 | 35,071,738 | 31,883,717 | 90.91% | 1 |
| 20 | 9 | 36,628,436 | 33,661,532 | 91.90% | 0.95 |
| 21 | 9.6 | 27,863,618 | 25,004,810 | 89.74% | 1 |
| 22 | 10 | 35,641,976 | 31,404,145 | 88.11% | 1 |
| 23 | 9.8 | 34,509,804 | 31,362,510 | 90.88% | 1 |
| 24 | 9.9 | 34,951,001 | 29,002,341 | 82.98% | 1 |
| 25 | 7.6 | 37,845,052 | 34,298,970 | 90.63% | 1 |
| 26 | 10 | 23,422,113 | 21,276,648 | 90.84% | 1 |
| 27 | 9.9 | 38,833,918 | 35,039,844 | 90.23% | 1 |
| 28 | 9.8 | 31,433,349 | 26,756,067 | 85.12% | 1 |
| 29 | 8.6 | 33,280,602 | 30,544,937 | 91.78% | 1 |
| 30 | 9.6 | 28,382,694 | 25,572,807 | 90.10% | 1 |
| 31 | 9.5 | 22,649,348 | 18,108,154 | 79.95% | 1 |
| 32 | 9.9 | 24,287,675 | 22,016,777 | 90.65% | 1 |
| 33 | 9.9 | 26,686,979 | 22,385,038 | 83.88% | 0.98 |
| 34 | 7 | 35,060,443 | 31,375,591 | 89.49% | 1 |
| 35 | 9.9 | 29,824,176 | 26,400,361 | 88.52% | 1 |
| 36 | 8.6 | 25,699,324 | 23,188,500 | 90.23% | 1 |
| 37 | 9.6 | 23,430,905 | 21,521,286 | 91.85% | 1 |
| 38 | 9.3 | 39,062,764 | 35,804,930 | 91.66% | 1 |
| 39 | 9.6 | 26,171,523 | 20,769,721 | 79.36% | 1 |
| 40 | 9.8 | 31,411,345 | 24,745,858 | 78.78% | 1 |
| 41 | 9.5 | 35,474,123 | 32,331,115 | 91.14% | 0.85 |
| 42 | 9.9 | 38,053,227 | 34,875,783 | 91.65% | 1 |
| 43 | 9.9 | 35,221,926 | 29,434,964 | 83.57% | 1 |
| 44 | 9.7 | 29,190,726 | 24,108,621 | 82.59% | 1 |
| 45 | 9.9 | 32,953,640 | 30,086,674 | 91.30% | 1 |
| 46 | 9.7 | 36,443,429 | 32,904,772 | 90.29% | 1 |
| 47 | 9.4 | 31,172,529 | 28,572,740 | 91.66% | 1 |
| 48 | 9.6 | 29,733,423 | 26,914,694 | 90.52% | 1 |
| 49 | 9 | 20,877,732 | 18,163,627 | 87.00% | 1 |
| 50 | 9.6 | 27,944,571 | 25,038,336 | 89.60% | 1 |
| 51 | 9.5 | 24,603,471 | 22,305,507 | 90.66% | 1 |
| 52 | 9.5 | 35,410,401 | 29,886,378 | 84.40% | 1 |
| 53 | 8.9 | 35,416,628 | 28,212,886 | 79.66% | 1 |
| 54 | 9.5 | 25,293,191 | 22,682,934 | 89.68% | 1 |
| 55 | 9.5 | 29,078,886 | 25,327,710 | 87.10% | 1 |
| 56 | 9.7 | 30,839,425 | 27,712,307 | 89.86% | 0.97 |
| 57 | 9.6 | 20,652,784 | 15,644,484 | 75.75% | 0.99 |
| 58 | 9.9 | 36,244,838 | 32,518,869 | 89.72% | 1 |
| 59 | 9.6 | 24,237,971 | 22,119,572 | 91.26% | 0.94 |
| 60 | 9.8 | 28,164,424 | 23,959,476 | 85.07% | 1 |
| 61 | 9.8 | 34,223,863 | 30,849,390 | 90.14% | 1 |
| 62 | 9.6 | 35,195,026 | 32,337,190 | 91.88% | 1 |
| 63 | 9.5 | 26,781,765 | 24,274,992 | 90.64% | 1 |
| 64 | 9.8 | 27,718,019 | 25,373,074 | 91.54% | 1 |
| 65 | 9.8 | 35,455,344 | 31,895,628 | 89.96% | 0.99 |
| 66 | 10 | 25,929,365 | 21,617,312 | 83.37% | 0.97 |
| 67 | 8.3 | 39,768,598 | 36,257,031 | 91.17% | 1 |
| 68 | 8.6 | 24,566,805 | 20,930,918 | 85.20% | 0.99 |
| 69 | 9.5 | 27,859,795 | 25,452,709 | 91.36% | 1 |
| 70 | 9.5 | 24,903,400 | 22,340,840 | 89.71% | 0.99 |
| 71 | 9.9 | 25,255,562 | 19,727,119 | 78.11% | 1 |
| 72 | 9.9 | 33,886,018 | 28,260,939 | 83.40% | 0.98 |
| 73 | 9.8 | 25,675,377 | 23,554,591 | 91.74% | 0.99 |
| 74 | 9.7 | 25,268,650 | 22,185,875 | 87.80% | 0.99 |
| 75 | 9.7 | 35,417,930 | 31,475,914 | 88.87% | 1 |
| 76 | 9.8 | 32,334,249 | 29,375,665 | 90.85% | 1 |
| 77 | 9.6 | 36,831,023 | 32,930,618 | 89.41% | 1 |
| 78 | 9.9 | 31,384,449 | 28,591,233 | 91.10% | 1 |
| 79 | 7.9 | 37,903,559 | 31,168,096 | 82.23% | 1 |
| 80 | 9.7 | 38,119,049 | 34,070,806 | 89.38% | 1 |
| 81 | 9.8 | 31,020,523 | 27,937,083 | 90.06% | 1 |
| 82 | 9.7 | 22,498,851 | 20,566,199 | 91.41% | 1 |
| 83 | 9.8 | 27,554,973 | 25,339,553 | 91.96% | 0.99 |
| 84 | 9.5 | 29,148,975 | 24,397,692 | 83.70% | 1 |
| 85 | 9.2 | 37,334,843 | 34,176,315 | 91.54% | 1 |
| 86 | 9.7 | 25,460,761 | 22,886,678 | 89.89% | 1 |
| 87 | 9.7 | 20,805,415 | 19,084,807 | 91.73% | 1 |
| 88 | 9.6 | 38,185,503 | 34,569,336 | 90.53% | 1 |
| 89 | 9.8 | 22,189,559 | 20,081,551 | 90.50% | 1 |
| 90 | 9.8 | 23,719,803 | 19,001,934 | 80.11% | 1 |
| 91 | 9.6 | 39,547,924 | 36,162,621 | 91.44% | 1 |
| 92 | 9.6 | 20,260,935 | 16,615,993 | 82.01% | 1 |
| 93 | 9.7 | 38,991,673 | 34,944,338 | 89.62% | 1 |
| 94 | 9.6 | 38,287,025 | 34,707,189 | 90.65% | 1 |
| 95 | 9.3 | 34,607,618 | 31,001,504 | 89.58% | 1 |
| 96 | 9.7 | 22,121,808 | 18,697,352 | 84.52% | 1 |
| 97 | 8.4 | 34,829,549 | 31,168,964 | 89.49% | 1 |
| 98 | 9.7 | 32,957,353 | 29,605,590 | 89.83% | 1 |
| 99 | 9.9 | 20,278,845 | 18,601,785 | 91.73% | 0.93 |
| 100 | 9.6 | 28,659,618 | 24,830,693 | 86.64% | 1 |
| 101 | 9.5 | 37,738,438 | 33,983,464 | 90.05% | 0.99 |
| 102 | 9.5 | 26,323,027 | 23,903,941 | 90.81% | 1 |
| 103 | 9.8 | 21,412,771 | 19,399,970 | 90.60% | 1 |
| 104 | 9.6 | 39,536,831 | 36,322,487 | 91.87% | 1 |
| 105 | 9.7 | 27,783,963 | 24,027,572 | 86.48% | 0.99 |
| 106 | 9.8 | 23,714,578 | 21,456,950 | 90.48% | 1 |
| 107 | 8.4 | 27,141,586 | 24,560,422 | 90.49% | 0.97 |
| 108 | 8.9 | 32,154,186 | 29,353,556 | 91.29% | 1 |
| 109 | 9.9 | 35,131,538 | 32,208,594 | 91.68% | 1 |
| 110 | 9.8 | 32,888,071 | 29,645,307 | 90.14% | 0.98 |
| 111 | 9.9 | 20,892,469 | 18,799,043 | 89.98% | 0.98 |
| 112 | 9.8 | 27,390,142 | 24,719,604 | 90.25% | 1 |
| 113 | 9.7 | 34,219,049 | 30,715,018 | 89.76% | 1 |
| 114 | 9.8 | 27,752,462 | 25,465,659 | 91.76% | 1 |
| 115 | 9.5 | 35,019,996 | 31,595,041 | 90.22% | 1 |
| 116 | 9.6 | 33,494,498 | 30,265,628 | 90.36% | 1 |
| 117 | 8.9 | 39,555,241 | 36,121,846 | 91.32% | 1 |
| 118 | 9.7 | 26,335,587 | 23,733,631 | 90.12% | 1 |
| 119 | 9.9 | 38,848,593 | 35,631,929 | 91.72% | 0.99 |
| 120 | 9.8 | 24,674,202 | 22,088,346 | 89.52% | 1 |
| 121 | 9.4 | 26,812,453 | 24,251,864 | 90.45% | 1 |
| 122 | 9.9 | 27,853,656 | 25,569,657 | 91.80% | 0.98 |
| 123 | 9.8 | 32,643,033 | 27,002,317 | 82.72% | 1 |
| 124 | 9.5 | 23,097,259 | 21,048,532 | 91.13% | 1 |
| 125 | 9.8 | 37,241,148 | 31,368,219 | 84.23% | 1 |
| 126 | 9.9 | 24,294,754 | 21,906,580 | 90.17% | 1 |
| 127 | 9.7 | 21,482,654 | 19,353,723 | 90.09% | 1 |
| 128 | 9.7 | 30,857,293 | 27,953,622 | 90.59% | 1 |
| 129 | 9 | 38,026,805 | 34,110,044 | 89.70% | 1 |
| 130 | 9.2 | 29,107,244 | 26,566,182 | 91.27% | 0.99 |
| 131 | 9.7 | 35,923,273 | 32,280,653 | 89.86% | 1 |
| 132 | 9.8 | 28,409,045 | 25,409,050 | 89.44% | 1 |
| 133 | 9.6 | 25,434,116 | 22,893,248 | 90.01% | 1 |
| 134 | 8.1 | 27,384,890 | 21,765,510 | 79.48% | 1 |
| 135 | 9.5 | 23,381,259 | 20,215,436 | 86.46% | 1 |
| 136 | 9.7 | 21,767,419 | 19,649,449 | 90.27% | 1 |
| 137 | 9.7 | 39,302,936 | 35,604,530 | 90.59% | 1 |
| 138 | 9.7 | 25,573,023 | 23,314,925 | 91.17% | 1 |
| 139 | 8.8 | 26,138,262 | 23,712,632 | 90.72% | 1 |
| 140 | 9.8 | 38,610,914 | 33,274,885 | 86.18% | 1 |
| 141 | 9.8 | 23,328,093 | 20,803,993 | 89.18% | 1 |
| 142 | 9.9 | 32,678,548 | 29,302,854 | 89.67% | 1 |
| 143 | 9.8 | 31,929,122 | 29,253,462 | 91.62% | 1 |
| 144 | 9.6 | 23,825,418 | 21,685,895 | 91.02% | 1 |
| 145 | 9.4 | 26,937,319 | 24,133,144 | 89.59% | 1 |
| 146 | 9.2 | 29,164,310 | 26,536,605 | 90.99% | 0.98 |
| 147 | 9.7 | 28,936,821 | 25,921,604 | 89.58% | 0.71 |
| 148 | 8.8 | 26,521,555 | 23,922,442 | 90.20% | 1 |
| 149 | 9.7 | 31,890,882 | 28,548,717 | 89.52% | 1 |
| 150 | 9.7 | 21,662,684 | 19,901,508 | 91.87% | 1 |
| 151 | 9.8 | 36,418,563 | 33,395,822 | 91.70% | 1 |
| 152 | 9.8 | 20,907,671 | 19,218,331 | 91.92% | 1 |
| 153 | 9.8 | 27,271,836 | 24,610,105 | 90.24% | 1 |
| 154 | 9.8 | 22,403,323 | 20,599,856 | 91.95% | 1 |
| 155 | 10 | 36,920,527 | 33,645,677 | 91.13% | 1 |
| 156 | 9.8 | 24,733,017 | 22,136,050 | 89.50% | 0.99 |
| 157 | 10 | 31,140,208 | 28,076,012 | 90.16% | 0.99 |
| 158 | 9.7 | 36,163,644 | 30,540,197 | 84.45% | 1 |
