# Supplementary material for: Genome-wide profiling of DNA methylome and transcriptome in peripheral blood monocytes for major depression: A Monozygotic Discordant Twin Study
Source: Transl Psychiatry. 2019 Sep 2;9:215. doi: 10.1038/s41398-019-0550-2 (PMC6718674; doi:10.1038/s41398-019-0550-2)
Supplement: Supplementary file 10 — Table S2 [file 41398_2019_550_MOESM10_ESM.docx]

**Table S2**. Significant CpG probes replicated in the brain (same direction)

| Region ID | Probe | Gene | Chr | Position | FC | P^a^ | q |
| --- | --- | --- | --- | --- | --- | --- | --- |
| 1 | cg09895920 | *CREB3L4* | 1 | 153,941,186 | 1.27 | 1.07×10^-2^ | 3.21×10^-2^ |
| 2 | cg24563094 | *GAREML* | 2 | 26,395,458 | 1.14 | 1.44×10^-3^ | 7.23×10^-3^ |
| 2 | cg22470850 | *GAREML* | 2 | 26,395,824 | 1.13 | 1.77×10^-3^ | 8.85×10^-3^ |
| 3 | cg19283506 | *CHST10* | 2 | 101,034,270 | 1.14 | 2.87×10^-3^ | 3.44×10^-2^ |
| 4 | cg03902565 | *NNT* | 5 | 43,603,176 | 1.12 | 2.81×10^-3^ | 2.25×10^-2^ |
| 5 | cg03249630 | *ANKRD22* | 10 | 90,611,782 | 1.18 | 4.26×10^-3^ | 2.98×10^-2^ |
| 5 | cg01561719 | *ANKRD22* | 10 | 90,611,855 | 1.23 | 4.46×10^-4^ | 3.12×10^-3^ |
| 5 | cg03818395 | *ANKRD22* | 10 | 90,612,228 | 1.10 | 7.10×10^-3^ | 4.26×10^-2^ |
| 6 | cg18395636 | *RAB38* | 11 | 87,908,785 | 1.20 | 1.31×10^-3^ | 1.05×10^-2^ |
| 7 | cg11549417 | *RASA3* | 13 | 114,814,643 | 1.23 | 1.35×10^-3^ | 9.45×10^-3^ |
| 8 | cg04202511 | *NFATC3* | 16 | 68,117,991 | 1.17 | 2.45×10^-3^ | 2.94×10^-2^ |
| 8 | cg17125623 | *NFATC3* | 16 | 68,119,985 | 1.46 | 2.74×10^-3^ | 1.64×10^-2^ |
| 9 | cg17628249 | *C17orf64* | 17 | 58,499,854 | 1.22 | 6.58×10^-4^ | 6.58×10^-3^ |
| 9 | cg02172058 | *C17orf64* | 17 | 58,499,911 | 1.24 | 2.63×10^-5^ | 1.58×10^-4^ |
| 10 | cg16007279 | *MAFF* | 22 | 38,598,948 | 1.15 | 4.43×10^-4^ | 3.10×10^-3^ |
| 10 | cg09035736 | *MAFF* | 22 | 38,599,166 | 1.21 | 4.44×10^-4^ | 1.78×10^-3^ |

^a^Adjusted for age and gender
