# Supplementary material for: Genome-wide profiling of DNA methylome and transcriptome in peripheral blood monocytes for major depression: A Monozygotic Discordant Twin Study
Source: Transl Psychiatry. 2019 Sep 2;9:215. doi: 10.1038/s41398-019-0550-2 (PMC6718674; doi:10.1038/s41398-019-0550-2)
Supplement: Supplementary file 11 — Table S3 [file 41398_2019_550_MOESM11_ESM.docx]

**Table S3**. Replication of differentially expressed genes (DEGs) in the brain

|  | Chr | Start (bp) | End (bp) | Nearest gene | FC^a^ | P^b^ | Direction with blood |
| --- | --- | --- | --- | --- | --- | --- | --- |
|  | 5 | 71,197,646 | 71,208,130 | *GUSBP9* | 0.28 | 1.52E-04 | N |
|  | 9 | 122,144,058 | 122,159,819 | *NDUFA8* | 0.03 | 4.22E-04 | Y |

^a^ Fold change (FC) in gene expression level between depressed twins and their non-depressed co-twins.

^b^ Adjusted for age, sex, RIN and brain PH.
