# Supplementary material for: Genome-wide profiling of DNA methylome and transcriptome in peripheral blood monocytes for major depression: A Monozygotic Discordant Twin Study
Source: Transl Psychiatry. 2019 Sep 2;9:215. doi: 10.1038/s41398-019-0550-2 (PMC6718674; doi:10.1038/s41398-019-0550-2)
Supplement: Supplementary file 12 — Table S4 [file 41398_2019_550_MOESM12_ESM.docx]

**Table S4.** List of significant correlation pairs between DNA methylation and *cis*-acting gene expression in peripheral blood monocytes

| Gene | Chr | DNA Methylation | | Expression | | Correlation | P**^a^** |
| --- | --- | --- | --- | --- | --- | --- | --- |
|  |  | Probe | Position (bp) | Start (bp) | End (bp) |  |  |
| *TAL1* | 1 | cg06463365 | 47,697,733 | 47,700,160 | 47,703,358 | -0.88 | 1.40×10^-6^ |
| *SLC25A25* | 9 | cg07688412 | 130,830,096 | 130,834,219 | 130,837,486 | 0.87 | 1.70×10^-6^ |
| *SH3GL3* | 15 | cg27648738 | 84,115,811 | 84,117,052 | 84,121,779 | 0.96 | 3.20×10^-6^ |
| *ZEB2* | 2 | cg03424727 | 145,277,646 | 145,278,318 | 145,284,285 | -0.96 | 5.40×10^-6^ |
| *SRI* | 7 | cg06737937 | 87,849,496 | 87,851,947 | 87,856,642 | -0.93 | 6.30×10^-6^ |
| *RPGRIP1L* | 16 | cg26746331 | 53,737,506 | 53,738,259 | 53,745,820 | 0.85 | 7.10×10^-6^ |
| *TAL1* | 1 | cg01418261 | 47,697,663 | 47,700,160 | 47,703,358 | -0.95 | 7.20×10^-6^ |
| *MAGI2* | 7 | cg19591626 | 78,400,561 | 78,405,293 | 78,408,797 | -0.9 | 8.00×10^-6^ |
| *NDRG4* | 16 | cg17650822 | 58,497,795 | 58,500,176 | 58,508,125 | 0.96 | 8.40×10^-6^ |
| *VPS37D* | 7 | cg13662144 | 73,082,340 | 73,084,835 | 73,089,169 | 0.93 | 8.70×10^-6^ |
| *TAL1* | 1 | cg19918343 | 47,697,673 | 47,700,160 | 47,703,358 | -0.96 | 8.80×10^-6^ |
| *NNT* | 5 | cg08052882 | 43,602,666 | 43,604,225 | 43,611,261 | 0.95 | 1.03×10^-5^ |
| *SPRED2* | 2 | cg14480116 | 65,594,890 | 65,596,164 | 65,603,423 | -0.85 | 1.03×10^-5^ |
|  | 11 | cg07211140 | 32,455,025 | 32,455,005 | 32,461,579 | 0.93 | 1.13×10^-5^ |
| *MAFF* | 22 | cg07207286 | 38,598,880 | 38,601,665 | 38,607,349 | 0.96 | 1.22×10^-5^ |
| *RAB1B* | 11 | cg15615396 | 66,035,392 | 66,035,869 | 66,040,194 | 0.94 | 1.29×10^-5^ |
| *KDM2B* | 12 | cg15234492 | 122,019,076 | 122,021,012 | 122,025,297 | 0.86 | 1.34×10^-5^ |
|  | 5 | cg22464292 | 140,777,446 | 140,778,318 | 140,783,255 | 0.93 | 1.38×10^-5^ |
| *N4BP2L2* | 13 | cg17936564 | 33,113,331 | 33,114,268 | 33,121,145 | 0.88 | 1.46×10^-5^ |
| *KLC2* | 11 | cg03128921 | 66,035,086 | 66,035,869 | 66,040,194 | 0.93 | 1.46×10^-5^ |
| *ZNF212* | 7 | cg12695158 | 148,936,883 | 148,937,822 | 148,943,315 | -0.95 | 1.58×10^-5^ |
| *RPGRIP1L* | 16 | cg26692097 | 53,738,201 | 53,738,259 | 53,745,820 | -0.91 | 1.66×10^-5^ |
| *NRXN3* | 14 | cg05468833 | 79,745,664 | 79,747,186 | 79,752,834 | 0.93 | 1.68×10^-5^ |
| *LRRC45* | 17 | cg11040439 | 79,980,929 | 79,981,680 | 79,985,887 | -0.87 | 1.72×10^-5^ |
| *CREB3L4* | 1 | cg01387743 | 153,940,674 | 153,942,761 | 153,949,543 | -0.95 | 1.73×10^-5^ |
| *PCDHGA11* | 5 | cg18118262 | 140,800,424 | 140,778,318 | 140,783,255 | 0.88 | 1.75×10^-5^ |
| *RP11* | 5 | cg18371052 | 8,457,721 | 8,460,078 | 8,466,246 | -0.9 | 1.79×10^-5^ |
| *SH3GL3* | 15 | cg22946150 | 84,116,107 | 84,117,052 | 84,121,779 | 0.91 | 1.80×10^-5^ |
| *CTD* | 5 | cg01817364 | 43,037,411 | 43,038,634 | 43,043,702 | 0.9 | 1.85×10^-5^ |
| *UFC1* | 1 | cg00939106 | 161,123,698 | 161,125,610 | 161,129,715 | 0.88 | 1.95×10^-5^ |
| *MAGI2* | 7 | cg16678001 | 78,400,383 | 78,405,293 | 78,408,797 | -0.85 | 2.11×10^-5^ |
| *FAM20B* | 1 | cg00562731 | 178,995,133 | 178,996,577 | 179,000,118 | 0.96 | 2.11×10^-5^ |
| *NFATC3* | 16 | cg07026259 | 68,119,185 | 68,119,586 | 68,124,112 | 0.92 | 2.28×10^-5^ |
| *RP11* | 15 | cg24750854 | 69,222,903 | 69,224,060 | 69,229,440 | 0.94 | 2.30×10^-5^ |
| *HIST1H2BI* | 6 | cg04704193 | 26,272,200 | 26,273,038 | 26,280,028 | 0.93 | 2.50×10^-5^ |
| *ZBTB45* | 19 | cg11457695 | 59,030,948 | 59,032,251 | 59,039,415 | 0.94 | 2.60×10^-5^ |
| *ZFP64* | 20 | cg20182785 | 50,722,303 | 50,723,020 | 50,730,004 | 0.91 | 2.76×10^-5^ |
| *RP11* | 5 | cg18394854 | 8,457,818 | 8,460,078 | 8,466,246 | -0.89 | 2.83×10^-5^ |
| *SORBS2* | 4 | cg12066473 | 186,733,331 | 186,734,615 | 186,742,456 | 0.92 | 2.88×10^-5^ |
| *CTD* | 16 | cg08346731 | 82,204,172 | 82,205,361 | 82,210,598 | -0.94 | 2.91×10^-5^ |
| *FRMD4B* | 3 | cg18433615 | 69,435,504 | 69,436,020 | 69,441,438 | 0.86 | 2.93×10^-5^ |
| *AC092431.1* | 2 | cg22629907 | 69,871,140 | 65,596,164 | 65,603,423 | 0.95 | 2.95×10^-5^ |
| *FANCC* | 9 | cg10862471 | 98,079,646 | 98,084,022 | 98,090,465 | 0.87 | 3.10×10^-5^ |
| *ACTL6A* | 3 | cg03839554 | 179,280,332 | 179,281,338 | 179,284,744 | 0.86 | 3.12×10^-5^ |
| *CTD* | 5 | cg23810282 | 43,037,519 | 43,038,634 | 43,043,702 | 0.95 | 3.14×10^-5^ |
| *RP11* | 5 | cg24581226 | 8,457,970 | 8,460,078 | 8,466,246 | -0.94 | 3.15×10^-5^ |
| *SLC25A11* | 17 | cg03889382 | 4,842,765 | 4,844,332 | 4,851,923 | -0.85 | 3.26×10^-5^ |
| *KIAA0513* | 16 | cg06276064 | 85,096,632 | 85,097,705 | 85,101,296 | 0.86 | 3.49×10^-5^ |
| *FAM59B* | 2 | cg17129645 | 26,395,833 | 26,397,782 | 26,404,786 | -0.84 | 3.51×10^-5^ |
| *N4BP2L2* | 13 | cg21921456 | 33,113,032 | 33,114,268 | 33,121,145 | 0.91 | 3.58×10^-5^ |
| *SWSAP1* | 19 | cg08405405 | 11,485,325 | 11,487,244 | 11,492,308 | 0.96 | 3.65×10^-5^ |
| *TAL1* | 1 | cg11766986 | 47,697,550 | 47,700,160 | 47,703,358 | -0.91 | 3.67×10^-5^ |
| *AC002456.2* | 7 | cg25757472 | 90,224,583 | 90,227,038 | 90,230,173 | 0.87 | 3.69×10^-5^ |
| *MYO1C* | 17 | cg03079497 | 1,390,554 | 1,393,069 | 1,397,501 | 0.89 | 3.80×10^-5^ |
|  | 6 | cg19147015 | 30,297,941 | 30,299,839 | 30,303,837 | 0.74 | 3.88×10^-5^ |
| *STRA13* | 17 | cg15578811 | 79,981,292 | 79,981,680 | 79,985,887 | 0.84 | 3.94×10^-5^ |
| *C17orf64* | 17 | cg12131208 | 58,499,700 | 58,472,476 | 58,478,500 | 0.96 | 3.96×10^-5^ |
| *STRA13* | 17 | cg25953504 | 79,981,121 | 79,981,680 | 79,985,887 | 0.84 | 3.96×10^-5^ |
| *AC019181.2* | 2 | cg20557037 | 165,698,099 | 165,700,427 | 165,704,462 | 0.93 | 3.97×10^-5^ |
| *N4BP2L2* | 13 | cg11630632 | 33,113,343 | 33,114,268 | 33,121,145 | 0.86 | 4.01×10^-5^ |
| *RAB38* | 11 | cg17108629 | 87,908,805 | 85,781,097 | 85,788,010 | 0.93 | 4.07×10^-5^ |
| *NRXN3* | 14 | cg09260207 | 79,746,520 | 79,747,186 | 79,752,834 | 0.96 | 4.21×10^-5^ |
| *ZBTB45* | 19 | cg26634707 | 59,030,662 | 59,032,251 | 59,039,415 | 0.9 | 4.28×10^-5^ |
| *ZNF212* | 7 | cg07704585 | 148,936,630 | 148,937,822 | 148,943,315 | -0.85 | 4.37×10^-5^ |
| *NNT* | 5 | cg08420334 | 43,603,343 | 43,604,225 | 43,611,261 | 0.87 | 4.38×10^-5^ |
| *NNT* | 5 | cg00452016 | 43,603,138 | 43,604,225 | 43,611,261 | 0.94 | 4.51×10^-5^ |
| *STRA13* | 17 | cg04875987 | 79,981,264 | 79,981,680 | 79,985,887 | 0.94 | 4.51×10^-5^ |
| *FAM20B* | 1 | cg06528214 | 178,995,107 | 178,996,577 | 179,000,118 | 0.88 | 4.89×10^-5^ |
| *HSPB11* | 1 | cg15513671 | 54,412,007 | 54,414,946 | 54,420,001 | 0.85 | 4.90×10^-5^ |
| *C17orf64* | 17 | cg06752482 | 58,499,816 | 58,472,476 | 58,478,500 | 0.94 | 4.91×10^-5^ |
|  | 11 | cg27409910 | 32,454,216 | 32,455,005 | 32,461,579 | 0.8 | 5.12×10^-5^ |
| *HTRA4* | 8 | cg21184369 | 38,831,148 | 38,832,480 | 38,837,576 | 0.89 | 5.24×10^-5^ |
| *TMEM194A* | 12 | cg21721432 | 57,472,784 | 57,474,160 | 57,477,980 | 0.86 | 5.25×10^-5^ |
| *FRMD4B* | 3 | cg19522075 | 69,435,780 | 69,436,020 | 69,441,438 | 0.84 | 5.40×10^-5^ |
| *HSPA13* | 21 | cg01662102 | 15,755,986 | 15,758,849 | 15,764,866 | 0.87 | 5.46×10^-5^ |
| *FTO* | 16 | cg18821731 | 53,737,871 | 53,738,259 | 53,745,820 | -0.91 | 5.48×10^-5^ |
| *NFATC3* | 16 | cg07981599 | 68,119,049 | 68,119,586 | 68,124,112 | 0.91 | 5.49×10^-5^ |
|  | 11 | cg25835307 | 87,908,134 | 85,781,097 | 85,788,010 | 0.89 | 5.66×10^-5^ |
| *STRA13* | 17 | cg25213539 | 79,981,084 | 79,981,680 | 79,985,887 | 0.91 | 5.81×10^-5^ |
| *KLC2* | 11 | cg15201417 | 66,034,922 | 66,035,869 | 66,040,194 | 0.96 | 5.83×10^-5^ |
| *PICALM* | 11 | cg16633848 | 85,780,144 | 85,781,097 | 85,788,010 | 0.96 | 5.84×10^-5^ |
| *VPS37D* | 7 | cg24954661 | 73,082,001 | 73,084,835 | 73,089,169 | 0.91 | 5.85×10^-5^ |
| *C16orf95* | 16 | cg10067538 | 87,351,824 | 87,352,557 | 87,358,880 | 0.88 | 5.88×10^-5^ |
| *SF3B3* | 16 | cg07751125 | 70,557,411 | 70,558,784 | 70,564,104 | 0.92 | 5.94×10^-5^ |
| *FAM20B* | 1 | cg05383153 | 178,995,099 | 178,996,577 | 179,000,118 | 0.95 | 5.97×10^-5^ |
| *FAM59B* | 2 | cg24563094 | 26,395,458 | 26,397,782 | 26,404,786 | -0.84 | 6.02×10^-5^ |
| *ZBTB45* | 19 | cg14212467 | 59,030,979 | 59,032,251 | 59,039,415 | 0.91 | 6.09×10^-5^ |
| *SLC25A11* | 17 | cg11432441 | 4,842,610 | 4,844,332 | 4,851,923 | -0.93 | 6.11×10^-5^ |
| *ZEB2* | 2 | cg19101754 | 145,277,381 | 145,278,318 | 145,284,285 | -0.92 | 6.11×10^-5^ |
| *USP32* | 17 | cg18654231 | 58,469,739 | 58,472,476 | 58,478,500 | 0.88 | 6.28×10^-5^ |
| *RAB1B* | 11 | cg02520768 | 66,035,485 | 66,035,869 | 66,040,194 | 0.94 | 6.35×10^-5^ |
| *CIRH1A* | 16 | cg00615892 | 69,166,530 | 68,119,586 | 68,124,112 | 0.96 | 6.41×10^-5^ |
| *RFC5* | 12 | cg00670756 | 118,454,418 | 118,457,394 | 118,460,794 | 0.84 | 6.47×10^-5^ |
| *RP11* | 5 | cg17877220 | 8,458,089 | 8,460,078 | 8,466,246 | -0.92 | 6.54×10^-5^ |
| *CTH* | 1 | cg02917772 | 70,876,623 | 70,878,838 | 70,884,286 | -0.85 | 6.54×10^-5^ |
| *AC002456.2* | 7 | cg26735135 | 90,224,886 | 90,227,038 | 90,230,173 | 0.86 | 6.54×10^-5^ |
| *SRI* | 7 | cg14644787 | 87,849,494 | 87,851,947 | 87,856,642 | -0.84 | 6.60×10^-5^ |
| *NNT* | 5 | cg12656077 | 43,602,605 | 43,604,225 | 43,611,261 | 0.85 | 6.65×10^-5^ |
| *ZBTB45* | 19 | cg17364234 | 59,031,070 | 59,032,251 | 59,039,415 | 0.88 | 6.70×10^-5^ |
| *CTD* | 5 | cg04268624 | 43,037,285 | 43,038,634 | 43,043,702 | 0.87 | 6.85×10^-5^ |
| *NAIF1* | 9 | cg16950519 | 130,829,748 | 130,834,219 | 130,837,486 | 0.94 | 6.86×10^-5^ |
| *C16orf95* | 16 | cg02223001 | 87,351,033 | 87,352,557 | 87,358,880 | 0.91 | 6.97×10^-5^ |
| *LRRC45* | 17 | cg04489846 | 79,980,949 | 79,981,680 | 79,985,887 | -0.9 | 6.99×10^-5^ |
| *SPRED2* | 2 | cg00376294 | 65,594,797 | 65,596,164 | 65,603,423 | -0.89 | 7.03×10^-5^ |
| *PICALM* | 11 | cg09030501 | 85,779,252 | 85,781,097 | 85,788,010 | 0.96 | 7.14×10^-5^ |
| *TMEM5* | 12 | cg06437928 | 64,173,769 | 64,175,881 | 64,180,729 | 0.84 | 7.17×10^-5^ |
| *SF3B3* | 16 | cg20435469 | 70,557,679 | 70,558,784 | 70,564,104 | 0.84 | 7.17×10^-5^ |
|  | 13 | cg23132774 | 114,814,171 | 114,815,864 | 114,819,100 | -0.74 | 7.21×10^-5^ |
| *NRXN3* | 14 | cg22908679 | 79,746,212 | 79,747,186 | 79,752,834 | 0.89 | 7.28×10^-5^ |
| *PCDHGA11* | 5 | cg26647197 | 140,800,398 | 140,778,318 | 140,783,255 | 0.93 | 7.33×10^-5^ |
| *KLC2* | 11 | cg14442997 | 66,035,267 | 66,035,869 | 66,040,194 | 0.84 | 7.39×10^-5^ |
| *SLC30A3* | 2 | cg10629682 | 27,486,061 | 27,488,436 | 27,495,664 | 0.91 | 7.53×10^-5^ |
| *RAB1B* | 11 | cg02351179 | 66,035,370 | 66,035,869 | 66,040,194 | 0.86 | 7.53×10^-5^ |
| *CTH* | 1 | cg03755098 | 70,876,598 | 70,878,838 | 70,884,286 | -0.91 | 7.65×10^-5^ |
| *MAFF* | 22 | cg16007279 | 38,598,948 | 38,601,665 | 38,607,349 | 0.95 | 7.76×10^-5^ |
| *NRXN3* | 14 | cg14335579 | 79,745,997 | 79,747,186 | 79,752,834 | 0.91 | 7.86×10^-5^ |
| *NDRG4* | 16 | cg08791131 | 58,497,801 | 58,500,176 | 58,508,125 | 0.94 | 8.03×10^-5^ |
| *STRA13* | 17 | cg17241816 | 79,981,086 | 79,981,680 | 79,985,887 | 0.93 | 8.09×10^-5^ |
| *NDRG4* | 16 | cg13031432 | 58,497,767 | 58,500,176 | 58,508,125 | 0.93 | 8.15×10^-5^ |
| *TAL1* | 1 | cg26939858 | 47,697,669 | 47,700,160 | 47,703,358 | -0.92 | 8.30×10^-5^ |
| *NNT* | 5 | cg13102118 | 43,602,505 | 43,604,225 | 43,611,261 | 0.89 | 8.38×10^-5^ |
| *RNF219* | 1 | cg12548634 | 44,884,109 | 44,885,172 | 44,890,475 | 0.85 | 8.52×10^-5^ |
| *TMEM5* | 12 | cg05228379 | 64,173,617 | 64,175,881 | 64,180,729 | 0.92 | 8.75×10^-5^ |
| *SPRED2* | 2 | cg10831427 | 65,594,760 | 65,596,164 | 65,603,423 | -0.89 | 8.75×10^-5^ |
| *ZWILCH* | 15 | cg17722664 | 66,797,429 | 66,800,359 | 66,807,961 | 0.91 | 8.81×10^-5^ |
| *PICALM* | 11 | cg02920502 | 85,780,029 | 85,781,097 | 85,788,010 | 0.86 | 9.02×10^-5^ |
| *LPAR2* | 19 | cg02362385 | 19,739,192 | 19,740,069 | 19,747,480 | 0.94 | 9.09×10^-5^ |
|  | 5 | cg04553690 | 140,777,501 | 140,778,318 | 140,783,255 | 0.93 | 9.12×10^-5^ |
|  | 11 | cg17428011 | 123,173,101 | 123,175,220 | 123,179,990 | -0.84 | 9.18×10^-5^ |
| *MYO1C* | 17 | cg02317299 | 1,390,182 | 1,393,069 | 1,397,501 | 0.85 | 9.23×10^-5^ |
| *FAM20B* | 1 | cg22332891 | 178,995,082 | 178,996,577 | 179,000,118 | 0.96 | 9.24×10^-5^ |
| *RP11* | 5 | cg04828267 | 8,457,538 | 8,460,078 | 8,466,246 | -0.94 | 9.44×10^-5^ |
| *RNF220* | 1 | cg16547629 | 44,884,131 | 44,885,172 | 44,890,475 | 0.92 | 9.56×10^-5^ |
| *AC019181.2* | 2 | cg26373663 | 165,698,219 | 165,700,427 | 165,704,462 | 0.94 | 9.58×10^-5^ |
| *NFATC3* | 16 | cg09049717 | 68,119,261 | 68,119,586 | 68,124,112 | 0.91 | 9.60×10^-5^ |
| *C16orf95* | 16 | cg01367424 | 87,351,490 | 87,352,557 | 87,358,880 | 0.86 | 9.64×10^-5^ |
| *KLC2* | 11 | cg10498476 | 66,035,147 | 66,035,869 | 66,040,194 | 0.95 | 9.68×10^-5^ |
| *CTH* | 1 | cg00968021 | 70,876,888 | 70,878,838 | 70,884,286 | -0.93 | 9.74×10^-5^ |
| *C17orf64* | 17 | cg02172058 | 58,499,911 | 58,472,476 | 58,478,500 | 0.86 | 9.95×10^-5^ |
| *KLC2* | 11 | cg01280128 | 66,034,963 | 66,035,869 | 66,040,194 | 0.86 | 9.97×10^-5^ |

**^a^**Calculated by permutation.
