# Supplementary material for: Genome-wide profiling of DNA methylome and transcriptome in peripheral blood monocytes for major depression: A Monozygotic Discordant Twin Study
Source: Transl Psychiatry. 2019 Sep 2;9:215. doi: 10.1038/s41398-019-0550-2 (PMC6718674; doi:10.1038/s41398-019-0550-2)
Supplement: Supplementary file 13 — Table S5 [file 41398_2019_550_MOESM13_ESM.docx]

**Table S5.** Co-methylation modules along with hub genes and biological pathways in each module

| \| Module \| Size \| P^a^ \| Top enriched pathway \| Hub gene \| Genes in the module \| \| --- \| --- \| --- \| --- \| --- \| --- \| \| 1 \| 167 \| 9.22×10^-8^ \| Neural nucleus development \| *SLC34A1* \| *SLC34A1, TTN, BNC2, VWA1, MAFF, TBC1D24, GPX8, RUSC2, MLEC, BCAN, HSPB11, SH2D4A, PIK3CA, PDZD3, DKC1, ARHGEF26, INPP5E, BNC1, ARID3B, NINJ2, PKMYT1, ZBTB5, KCNS3, RAD1, HLA-DOA, GPLD1, ACSS2, EIF4G2, ADNP, FAT3, UACA, SIM2, CDON, RBP1, RSPO4, CDH11, PIGY, AAK1, MAPRE2, USP6NL, PANX2, ADGRE2, TMEM33, FANCC, NKD2, TMCO3, PHOX2B, FAM207A, SWSAP1, HTT, ZFAT, GLP1R, DIRC3, CCDC129, NEDD8, BCL2L11, STK32B, DENND3, SLC25A25, BDKRB2, AXIN2, ZNF384, GPR97, KLF9, E2F2, UFC1, CUL1, TBC1D31, RNF4, PAQR5, CNDP2, LTF, ATAD5, KLHL8, MR1, SLC30A3, VPS37D, FKBP5, NCOR2, ACTL6A, ATL3, MGMT, CAPZB, CTH, FAM150B, JADE1, MOB2, INPP4B, TPM3, TMEM87B, CATSPERB, GLDN, TOX, RPP30, NTM, FAM98C, MAP2K5, GARS, PCDHGA11, ZNF212, SGSM3, LHX3, NOX5, ALDH1A3, CASC5, CLMN, MIPOL1, FXYD5, PYGM, TJP1, ARHGEF7, IGFBP4, ELMO1, ZNF679, FAM19A5, STK11IP, C22orf13, RP11, ZNF672, THADA, COL9A3, FTO, GDA, RFX1, PTRF, BOLA1, NPTX1, TTLL10, RAB3GAP1, SCN5A, SYDE1, PKD1L2, RNF8, SNX17, MAGI2, HNRNPA0, SORCS2, TRIB3, ADD1, NRXN3, CNIH3, BARX2, CDK14, CYB561, DGKQ, UNKL, AGAP1, MATN2, SHTN1, ZNF710, HMGXB3, GRIN1, TMEM5, BCL2L13, TM4SF20, VPS41, CHST10, PHF21B, PCYT2, HOXA9, OR2AG1, PRKD3, TESK2, KHSRP, EXOC4, RFC5, C9orf37* \| \| 2 \| 80 \| 2.17×10^-4^ \| Positive regulation of endothelial cell proliferation \| *MYO3B* \| *MYO3B, TMEM194B, PPID, SETD1B, BRE, PGM1, MAPK10, C6orf48, RASGRP4, SNCAIP, RAB7A, GIN1, PRSS21, PROX1, CDH13, STRIP2, RAB38, UBE3B, GRWD1, SORBS2, DOK4, SEC1P, PFN1, UTP15, ATP8A1, TSPO2, CCDC17, ANKRD11, TRIM39, WNT7B, SHROOM3, SFMBT2, BAT3, MIA3, FBXO42, GAREML, LAMA2, RDBP, EIF4E2, FAM161B, ABI2, DLK2, DDIT3, SP110, ELAC1, FOXI3, SORCS1, C17orf64, NCF1B, FRMD4B, ACKR2, STAC, IRGM, CHD4, SRSF10, IRAK3, JUND, GHR, KDM2B, MBD6, SYN3, KIRREL3, ADAD2, CELSR1, CREB3L4, TAL1, ENPP7, LRRC1, C19orf81, SF3B3, HAND1, KIAA0513, PGGT1B, E2F3, ADD2, HADH, F12, FOXD3, ZBTB4, ACTR5* \| \| 3 \| 57 \| 9.22×10^-8^ \| Cellular response to cAMP \| *CACNA1D* \| *CACNA1D, SNX24, ANKRD22, PICALM, FAM96B, CLVS1, CACNA1E, CCR6, C3orf75, ADAM32, NEMP1, CTDP1, GPR160, MAEA, ZNF585B, CCDC28A, MCF2L, ZNF618, RRN3, EDAR, PIK3CG, LOC286135, FAM20B, NNT, MADD, HMGB3, FUBP3, THSD1P, RAF1, HADHB, DRG2, CUTA, KCNQ1, SIDT2, LPAR2, RBM47, BHLHE40, ZBTB45, KREMEN1, R3HDM1, ARL15, MYOZ3, OSGIN2, ATE1, ALS2CL, CUX1, TBKBP1, CLIP2, FAM125B, SPRED2, ACAD9, KRI1, LIPE, BMP4, NFATC3, ZWILCH, GFI1* \| \| ^a^P-value for the association between MDD and the first three eigenvalues of a module \| \| \| \| \| \| |  |  |  |  |  |
| --- | --- | --- | --- | --- | --- | --- | --- | --- | --- | --- | --- | --- | --- | --- | --- | --- | --- | --- | --- | --- | --- | --- | --- | --- | --- | --- | --- | --- | --- | --- | --- | --- | --- | --- | --- |
