# Supplementary material for: Genome-wide profiling of DNA methylome and transcriptome in peripheral blood monocytes for major depression: A Monozygotic Discordant Twin Study
Source: Transl Psychiatry. 2019 Sep 2;9:215. doi: 10.1038/s41398-019-0550-2 (PMC6718674; doi:10.1038/s41398-019-0550-2)
Supplement: Supplementary file 14 — Table S6 [file 41398_2019_550_MOESM14_ESM.docx]

**Table S6.** Co-expression modules along with hub genes and biological pathways in each module

| \| Module \| Size \| P^a^ \| Top enriched pathway \| Hub gene \| Genes in the module \| \| --- \| --- \| --- \| --- \| --- \| --- \| \| 1 \| 94 \| 2.72×10^-5^ \| positive regulation of cytokine secretion \| *GPR34* \| *GPR34, ZNF304, GTF2E1, SRSF5, MGAT4A, SCARNA13, AC025259.1, RTP4, EIF4EP1, IER3, SVIL-AS1, ARHGAP24, DHRS4L2, CLEC5A, HPS5, ORM1, COL17A1, HIST1H3B, CALM2P2, PLK3, ADPRHL2, RN7SL752P, SNORD3B-2, HTRA1, CLEC1B, GIMAP8, AC087521.4, NEU1, AL032821.1, CCDC125, GABARAPL1, PMVK, PYGL, ZNF671, ZNF816, PGM1, TTN-AS1, TCTN3, PECR, SLC11A1, AC100810.1, KLF7-IT1, NT5C3B, AHR, UGCG, FAM43A, DGKD, SPSB2, SNORD46, ANKRD28, RABGEF1, AL583722.2, SNORA79, LINC01578, HYLS1, GPSM2, PSMD4, ZNF232, LTB4R, HIKESHI, RASGEF1B, GIMAP1-GIMAP5, DPCD, AKIRIN2, TTC9C, NFKBID, TRA2B, KBTBD11, TSPAN2, SNHG15, GIMAP1, LIN7A, RN7SL600P, ATP8A1, STAG3L3, RBBP5, MIR222HG, TRPT1, KNOP1, TMCO4, CIP2A, ATP1B1, ZKSCAN4, ST13, DNTTIP1, PAQR8, RPP25L, DDHD2, SNORA32, CALD1, RAB31, FADD, PAM, CD300A* \| \| 2 \| 62 \| 3.32×10^-5^ \| negative regulation of NF-kappaB transcription factor activity \| *MMAA* \| *MMAA, PLAC8, CD44-AS1, PIGA, MRPL27, TNFAIP8L2, AC245128.3, MR1, DHFR, CRTAM, AL627309.2, TTI2, SNORD3B-1, POLD1, MRPS26, AL132656.3, ZNF487, PFKFB2, SRGN, ZNF101, CYTIP, C3orf14, NUP50-AS1, TAF1B, GUSBP1, NDUFA8, ID1, TBC1D7, CYP4F3, RRP7A, AC092651.2, AC008993.1, YIPF4, TOMM7, ZNF223, MIR22HG, HSPBP1, CRIPT, KCNJ2, CBR1, PCNX2, DEFA3, HIST2H2AB, SIAH2, ARG1, RFK, ADHFE1, SEMA3C, ZNF627, OXSM, MIR181A1HG, AC020916.1, ABHD10, S100P, NAMPT, YOD1, ATP13A3, ZEB2-AS1, AC011472.2, PEX11B, CRYBB2P1, PIWIL4* \| \| 3 \| 53 \| 1.62×10^-5^ \| regulation of response to stress \| *RNF181* \| *RNF181, MRPL50, ZNF200, UAP1L1, FAR2, NME2, SNORA71E, EIF2S1, WASH5P, AC010761.1, CASP6, AP001372.3, NTHL1, CMTR2, MXRA7, RBBP6, MRPS18B, PPP1R10, RFLNB, LINC01003, AL121761.1, AC012368.1, PPID, BUD13, DBR1, MINPP1, ISOC2, CCT7, AC008038.1, ERVK9-11, USP41, PPM1G, F2RL1, TFF3, ZNF493, HPS6, RAPGEF6, DNASE1L1, UQCRFS1P1, BLM, ARL11, TMEM170B, SOWAHD, MRFAP1L1, PGP, SERPINB10, H1F0, ANKRD36B, AC087385.1, YPEL5, CDKN2D, AC115618.3, NECAP2* \| \| 4 \| 46 \| 9.40×10^-5^ \| regulation of neuron death \| *TCEANC* \| *TCEANC, CLTCL1, LYSMD2, TSPAN4, C16orf74, ANXA1, NDUFAF4, STAG3L5P, AL133342.1, AC139495.1, TNFSF10, ALG12, FIS1, LILRB4, TMCC1-AS1, FAM107B, NFKBIA, ETS2, BBS10, AC016876.2, RBL1, NAT1, SGK1, THUMPD3-AS1, CDCA7L, HP, GSTM4, VPS72, MFAP1, MRPL44, AL928970.1, PTGER2, MCL1, MRPL46, TEX2, YRDC, RGCC, GPANK1, HSPA1B, AC015967.2, FCER1A, DDX5, MYCT1, GUSBP3, MGAT5, AL158152.1* \| \| 5 \| 37 \| 5.02×10^-5^ \| regulation of response to stress \| *PHF23* \| *PHF23, SNORA72, IFT172, FIP1L1, PELI1, CD59, TRIM21, CSGALNACT1, SUCLA2, MTO1, HMGB3, FAM96B, NME4, HNRNPLL, ZNF772, GUSBP9, RMI1, ICA1, CLEC4E, AC037198.2, BORA, G0S2, AL133445.2, TAF8, RF02121, ITGB7, APIP, IGF2R, RN7SKP255, EPB41L2, MBOAT2, CAPN3, AC004492.1, C11orf54, MTERF3, MINCR, AC114878.1* \| |  |  |  |  |  |
| --- | --- | --- | --- | --- | --- | --- | --- | --- | --- | --- | --- | --- | --- | --- | --- | --- | --- | --- | --- | --- | --- | --- | --- | --- | --- | --- | --- | --- | --- | --- | --- | --- | --- | --- | --- | --- | --- | --- | --- | --- | --- |

^a^P-value for the association between MDD and the first three eigenvalues of a module
