# Supplementary material for: Genome-wide profiling of DNA methylome and transcriptome in peripheral blood monocytes for major depression: A Monozygotic Discordant Twin Study
Source: Transl Psychiatry. 2019 Sep 2;9:215. doi: 10.1038/s41398-019-0550-2 (PMC6718674; doi:10.1038/s41398-019-0550-2)
Supplement: Supplementary file 15 — Table S7 [file 41398_2019_550_MOESM15_ESM.docx]

**Table S7.** Pathway enrichment for putative DMRs with nominal associations with MDD (P<0.001)

| Term | Description | P^a^ | q^b^ |
| --- | --- | --- | --- |
| GO:0031098 | Stress-activated protein kinase signaling cascade | 2.90×10^-6^ | 4.65×10^-3^ |
| GO:0046627 | Negative regulation of insulin receptor signaling pathway | 3.29×10^-5^ | 2.63×10^-2^ |
| GO:0070302 | Regulation of stress-activated protein kinase signaling cascade | 4.79×10^-5^ | 2.56×10^-2^ |
| KEGG:hsa04150 | mTOR signaling pathway | 7.20×10^-5^ | 2.88×10^-2^ |
| GO:0043524 | Negative regulation of neuron apoptotic process | 7.72×10^-5^ | 2.47×10^-2^ |
| GO:0048011 | Nerve growth factor receptor signaling pathway | 1.49×10^-4^ | 3.96×10^-2^ |

^a^Obtained by permuting random loci matched by gene density using the DEPICT. ^b^Adjusted for 1,627 GO terms/ pathways.
