# Supplementary material for: Genome-wide profiling of DNA methylome and transcriptome in peripheral blood monocytes for major depression: A Monozygotic Discordant Twin Study
Source: Transl Psychiatry. 2019 Sep 2;9:215. doi: 10.1038/s41398-019-0550-2 (PMC6718674; doi:10.1038/s41398-019-0550-2)
Supplement: Supplementary file 16 — Table S8 [file 41398_2019_550_MOESM16_ESM.docx]

**Table S8.** Results for sensitivity analysis of the identified DMRs

| Chr | Start (bp) | End (bp) | Size (bp) | Nearest gene | # of probes | Model 1^a^ | | Model 2^b^ | | Model 3^c^ | |
| --- | --- | --- | --- | --- | --- | --- | --- | --- | --- | --- | --- |
|  |  |  |  |  |  | Peak P | Region P^§^ | Peak P | Region P^§^ | Peak P | Region P^§^ |
| 16 | 2,866,834 | 2,868,001 | 1,168 | *PRSS21* | 10 | 8.71×10^-3^ | 1.50×10^-4^ | 1.60×10^-3^ | 9.60×10^-4^ | 1.91×10^-4^ | 9.60×10^-4^ |
| 5 | 43,037,123 | 43,037,666 | 544 | *ANXA2R* | 7 | 8.98×10^-4^ | 3.50×10^-4^ | 2.00×10^-4^ | 1.60×10^-4^ | 3.62×10^-4^ | 1.60×10^-4^ |
| 1 | 54,411,017 | 54,412,009 | 993 | *HSPB11* | 18 | 1.39×10^-4^ | 1.10×10^-4^ | 2.38×10^-4^ | 3.40×10^-4^ | 1.28×10^-4^ | 3.40×10^-4^ |
| 2 | 69,870,526 | 69,871,424 | 899 | *AAK1* | 8 | 2.06×10^-5^ | 8.10×10^-4^ | 7.33×10^-5^ | 5.80×10^-4^ | 4.87×10^-4^ | 5.80×10^-4^ |
| 4 | 186,732,926 | 186,733,331 | 406 | *SORBS2* | 8 | 5.57×10^-5^ | 2.00×10^-5^ | 1.06×10^-4^ | 5.00×10^-5^ | 2.85×10^-5^ | 5.00×10^-5^ |
| 2 | 26,395,359 | 26,395,859 | 501 | *GAREML* | 8 | 3.01×10^-5^ | 1.13×10^-3^ | 7.74×10^-4^ | 1.38×10^-3^ | 8.55×10^-4^ | 1.38×10^-3^ |
| 5 | 140,800,398 | 140,800,983 | 586 | *PCDHGA11* | 10 | 1.37×10^-3^ | 7.00×10^-5^ | 3.90×10^-3^ | 8.00×10^-5^ | 7.11×10^-5^ | 8.00×10^-5^ |
| 1 | 153,940,616 | 153,941,285 | 670 | *CREB3L4* | 6 | 2.79×10^-5^ | 2.00×10^-5^ | 3.31×10^-5^ | 2.00×10^-5^ | 2.95×10^-5^ | 2.00×10^-5^ |
| 5 | 43,602,380 | 43,603,353 | 974 | *NNT* | 17 | 1.64×10^-3^ | 4.70×10^-4^ | 1.79×10^-3^ | 5.20×10^-4^ | 5.38×10^-4^ | 5.20×10^-4^ |
| 5 | 8,457,538 | 8,458,392 | 855 | *RP11* | 9 | 1.00×10^-4^ | 5.00×10^-5^ | 9.21×10^-4^ | 1.20×10^-4^ | 7.40×10^-5^ | 1.20×10^-4^ |
| 22 | 38,598,577 | 38,599,166 | 590 | *MAFF* | 9 | 5.94×10^-3^ | 1.57×10^-2^ | **7.76×10^-3^** | **9.62×10^-2^** | **1.82×10^-2^** | **9.62×10^-2^** |
| 12 | 64,173,610 | 64,174,367 | 758 | *TMEM5* | 9 | 1.52×10^-5^ | 2.29×10^-3^ | 1.99×10^-5^ | 2.06×10^-3^ | 2.16×10^-3^ | 2.06×10^-3^ |
| 19 | 11,484,448 | 11,485,452 | 1,005 | *SWSAP1* | 14 | 4.12×10^-4^ | 3.00×10^-5^ | 9.24×10^-4^ | 5.00×10^-5^ | 2.70×10^-5^ | 5.00×10^-5^ |
| 2 | 27,485,922 | 27,486,460 | 539 | *SLC30A3* | 8 | 2.15×10^-4^ | 1.27×10^-3^ | 2.90×10^-4^ | 6.35×10^-3^ | 1.23×10^-3^ | 6.35×10^-3^ |
| 2 | 101,034,246 | 101,034,295 | 50 | *CHST10* | 6 | 1.94×10^-5^ | 8.20×10^-4^ | 2.68×10^-5^ | 5.40×10^-4^ | 7.87×10^-4^ | 5.40×10^-4^ |
| 11 | 85,779,252 | 85,780,378 | 1,127 | *PICALM* | 10 | 6.84×10^-4^ | 3.95×10^-3^ | 1.71×10^-4^ | 5.92×10^-3^ | 3.00×10^-3^ | 5.92×10^-3^ |
| 13 | 114,814,024 | 114,814,401 | 378 | *CHAMP1* | 5 | 1.44×10^-4^ | 1.99×10^-3^ | 9.69×10^-4^ | 2.59×10^-3^ | 1.34×10^-3^ | 2.59×10^-3^ |
| 11 | 32,454,216 | 32,455,025 | 810 | *WT1* | 8 | 1.82×10^-3^ | 1.31×10^-3^ | 1.78×10^-3^ | 3.07×10^-3^ | 8.37×10^-4^ | 3.07×10^-3^ |
| 16 | 70,557,411 | 70,557,707 | 297 | *SF3B3* | 10 | 1.27×10^-3^ | 1.00×10^-3^ | 2.73×10^-4^ | 3.90×10^-3^ | 8.06×10^-4^ | 3.90×10^-3^ |
| 7 | 90,224,158 | 90,225,380 | 1,223 | *CDK14* | 11 | 7.20×10^-3^ | 5.08×10^-3^ | 3.23×10^-3^ | 1.18×10^-3^ | 4.14×10^-3^ | 1.18×10^-3^ |
| 11 | 87,908,134 | 87,908,805 | 672 | *RAB38* | 7 | 1.44×10^-3^ | 1.13×10^-3^ | 8.06×10^-3^ | 1.58×10^-3^ | 1.69×10^-3^ | 1.58×10^-3^ |
| 12 | 122,019,031 | 122,019,117 | 87 | *KDM2B* | 5 | 8.07×10^-4^ | 1.30×10^-4^ | 5.02×10^-4^ | 3.30×10^-4^ | 1.14×10^-4^ | 3.30×10^-4^ |
| 16 | 85,096,632 | 85,097,151 | 520 | *KIAA0513* | 5 | 1.17×10^-3^ | 7.30×10^-4^ | 4.66×10^-3^ | 9.10×10^-4^ | 8.06×10^-4^ | 9.10×10^-4^ |
| 2 | 65,594,021 | 65,595,186 | 1,166 | *SPRED2* | 6 | 3.40×10^-4^ | 1.80×10^-4^ | 9.06×10^-4^ | 8.70×10^-4^ | 1.62×10^-4^ | 8.70×10^-4^ |
| 9 | 98,079,646 | 98,080,622 | 977 | *FANCC* | 10 | 5.91×10^-5^ | 4.20×10^-4^ | 2.42×10^-5^ | 8.70×10^-4^ | 6.03×10^-4^ | 8.70×10^-4^ |
| 10 | 90,611,604 | 90,612,228 | 625 | *ANKRD22* | 7 | 1.62×10^-3^ | 1.20×10^-4^ | 2.40×10^-3^ | 3.30×10^-4^ | 2.11×10^-4^ | 3.30×10^-4^ |
| 5 | 140,777,344 | 140,777,655 | 312 | *PCDHA1* | 9 | 6.14×10^-5^ | 4.70×10^-4^ | 6.71×10^-4^ | 1.30×10^-4^ | 5.61×10^-4^ | 1.30×10^-4^ |
| 1 | 178,994,834 | 178,995,133 | 300 | *FAM20B* | 8 | 1.41×10^-3^ | 8.39×10^-3^ | 2.58×10^-3^ | 7.14×10^-3^ | 6.05×10^-3^ | 7.14×10^-3^ |
| 7 | 148,936,572 | 148,937,410 | 839 | *ZNF212* | 9 | 2.17×10^-4^ | 4.11×10^-3^ | 1.99×10^-4^ | 1.87×10^-3^ | 2.47×10^-3^ | 1.87×10^-3^ |
| 16 | 68,118,822 | 68,119,261 | 440 | *NFATC3* | 9 | 6.07×10^-4^ | 6.00×10^-5^ | 6.24×10^-4^ | 6.00×10^-5^ | 5.93×10^-5^ | 6.00×10^-5^ |
| 19 | 59,030,662 | 59,031,081 | 420 | *ZBTB45* | 7 | 1.85×10^-3^ | 5.98×10^-3^ | 4.58×10^-3^ | 9.12×10^-3^ | 9.11×10^-3^ | 9.12×10^-3^ |
| 16 | 87,351,006 | 87,351,824 | 819 | *C16orf95* | 10 | 3.14×10^-3^ | 4.71×10^-3^ | 5.38×10^-3^ | 1.62×10^-3^ | 6.51×10^-3^ | 1.62×10^-3^ |
| 17 | 58,499,300 | 58,500,186 | 887 | *C17orf64* | 9 | 2.04×10^-4^ | 1.30×10^-4^ | 5.77×10^-4^ | 7.70×10^-4^ | 9.25×10^-5^ | 7.70×10^-4^ |
| 19 | 19,739,060 | 19,739,414 | 355 | *LPAR2* | 8 | 4.01×10^-3^ | 3.02×10^-3^ | 2.17×10^-3^ | 1.29×10^-3^ | 3.04×10^-3^ | 1.29×10^-3^ |
| 3 | 179,280,056 | 179,280,746 | 691 | *ACTL6A* | 9 | 6.47×10^-3^ | 2.40×10^-4^ | 3.85×10^-3^ | 2.20×10^-4^ | 3.30×10^-4^ | 2.20×10^-4^ |
| 1 | 70,876,598 | 70,877,381 | 784 | *CTH* | 9 | 2.32×10^-4^ | 1.40×10^-4^ | 2.55×10^-4^ | 2.10×10^-4^ | 1.41×10^-4^ | 2.10×10^-4^ |
| 7 | 78,400,383 | 78,400,769 | 387 | *MAGI2* | 5 | 2.83×10^-3^ | 3.34×10^-3^ | 8.60×10^-3^ | 1.25×10^-3^ | 2.70×10^-3^ | 1.25×10^-3^ |
| 6 | 30,297,174 | 30,297,941 | 768 | *TRIM39* | 10 | 8.21×10^-4^ | 1.62×10^-3^ | 6.26×10^-4^ | 2.61×10^-3^ | 1.77×10^-3^ | 2.61×10^-3^ |
| 15 | 69,222,400 | 69,223,018 | 619 | *NOX5* | 7 | 1.18×10^-3^ | 1.20×10^-4^ | 1.71×10^-3^ | 2.30×10^-4^ | 1.73×10^-4^ | 2.30×10^-4^ |

^a^Model 1 further adjusted for childhood traumatic experience. ^b^Model 2 further adjusted for antidepressant usage. ^c^Model 3 further adjusted for use of antidepressants. ^§^Calculated by 100,000 times permutation.
