# Supplementary material for: Genome-wide profiling of DNA methylome and transcriptome in peripheral blood monocytes for major depression: A Monozygotic Discordant Twin Study
Source: Transl Psychiatry. 2019 Sep 2;9:215. doi: 10.1038/s41398-019-0550-2 (PMC6718674; doi:10.1038/s41398-019-0550-2)
Supplement: Supplementary file 17 — Table S9 [file 41398_2019_550_MOESM17_ESM.docx]

**Table S9.** Results for sensitivity analysis of the identified DEGs

| Chr | Start (bp) | End (bp) | Gene | Model 1^a^ | | Model 2^b^ | | Model 3^c^ | |
| --- | --- | --- | --- | --- | --- | --- | --- | --- | --- |
|  |  |  |  | FC | P | FC | P | FC | P |
| 2 | 215,996,329 | 216,082,955 | *PECR* | 1.52 | 1.64×10^-7^ | 1.52 | 4.30×10^-7^ | 1.96 | 2.26×10^-7^ |
| 1 | 185,292,384 | 185,294,372 | *AL356273.3* | 1.34 | 1.20×10^-6^ | 1.33 | 2.91×10^-6^ | 0.87 | 9.49×10^-7^ |
| 1 | 44,800,225 | 44,805,990 | *PLK3* | 2.55 | 5.73×10^-6^ | 2.41 | 7.09×10^-5^ | 1.50 | 1.00×10^-5^ |
| 5 | 71,197,646 | 71,208,130 | *GUSBP9* | 1.50 | 2.71×10^-6^ | 1.48 | 2.71×10^-5^ | 0.90 | 3.70×10^-6^ |
| 8 | 63,015,079 | 63,039,171 | *GGH* | 1.28 | 8.48×10^-6^ | 1.24 | 2.89×10^-5^ | 1.05 | 7.48×10^-6^ |
| 6 | 11,538,278 | 11,583,524 | *TMEM170B* | 8.15 | 5.44×10^-5^ | 7.89 | 8.35×10^-5^ | 10.20 | 3.79×10^-5^ |
| 11 | 64,223,799 | 64,226,254 | *TRPT1* | 0.75 | 1.10×10^-5^ | 0.77 | 2.90×10^-5^ | 1.21 | 1.28×10^-5^ |
| 16 | 19,701,934 | 19,718,235 | *KNOP1* | 0.78 | 7.19×10^-5^ | 0.77 | 2.64×10^-4^ | 0.44 | 1.10×10^-4^ |
| 16 | 3,222,325 | 3,236,221 | *ZNF200* | 0.82 | 2.65×10^-5^ | 0.80 | 5.49×10^-5^ | 0.64 | 2.38×10^-5^ |
| 6 | 30,617,709 | 30,626,395 | *MRPS18B* | 0.55 | 2.34×10^-4^ | 0.58 | 1.30×10^-3^ | 0.53 | 2.37×10^-4^ |
| 7 | 66,682,164 | 66,811,464 | *RABGEF1* | 1.44 | 8.02×10^-6^ | 1.43 | 3.77×10^-5^ | 0.86 | 5.44×10^-6^ |
| 22 | 49,900,229 | 49,918,458 | *ALG12* | 1.17 | 2.11×10^-5^ | 1.24 | 3.34×10^-5^ | 1.18 | 1.64×10^-5^ |
| 12 | 52,076,841 | 52,082,084 | *AC025259.1* | 0.73 | 2.07×10^-5^ | 0.74 | 1.84×10^-4^ | 0.60 | 1.90×10^-5^ |
| 19 | 19,668,796 | 19,683,509 | *ZNF101* | 0.58 | 3.05×10^-4^ | 0.61 | 2.08×10^-3^ | 0.48 | 4.41×10^-4^ |
| 1 | 151,156,629 | 151,159,749 | *TNFAIP8L2* | 0.16 | 1.82×10^-5^ | 0.17 | 1.47×10^-4^ | 0.13 | 1.86×10^-5^ |
| 8 | 33,473,386 | 33,513,601 | *TTI2* | 0.78 | 8.28×10^-5^ | 0.81 | 2.18×10^-4^ | 0.76 | 7.32×10^-5^ |
| 13 | 41,457,559 | 41,470,882 | *RGCC* | 3.99 | 6.17×10^-5^ | 4.05 | 2.83×10^-4^ | 4.29 | 4.70×10^-5^ |
| 9 | 122,144,058 | 122,159,819 | *NDUFA8* | 0.57 | 2.27×10^-5^ | 0.57 | 1.13×10^-4^ | 0.88 | 3.03×10^-5^ |
| 11 | 93,741,591 | 93,764,749 | *C11orf54* | 0.59 | 7.00×10^-5^ | 0.64 | 2.25×10^-4^ | 0.40 | 5.45×10^-5^ |
| 16 | 85,690,084 | 85,751,129 | *C16orf74* | 1.63 | 5.88×10^-4^ | 1.59 | 7.24×10^-3^ | 1.92 | 5.75×10^-4^ |
| 7 | 80,742,538 | 80,922,359 | *SEMA3C* | 1.76 | 6.33×10^-5^ | 1.85 | 2.42×10^-4^ | 1.21 | 3.91×10^-5^ |
| 19 | 21,397,119 | 21,427,573 | *ZNF493* | 1.64 | 4.55×10^-5^ | 1.65 | 6.90×10^-4^ | 1.24 | 5.29×10^-5^ |
| 11 | 4,384,897 | 4,393,696 | *TRIM21* | 0.26 | 4.60×10^-4^ | 0.28 | 3.39×10^-3^ | 0.22 | 4.81×10^-4^ |
| 19 | 52,949,379 | 52,962,911 | *ZNF816* | 0.74 | 4.29×10^-5^ | 0.77 | 9.93×10^-5^ | 0.60 | 3.04×10^-5^ |
| 10 | 43,436,841 | 43,483,179 | *ZNF487* | 1.36 | 5.60×10^-5^ | 1.27 | 1.60×10^-3^ | 2.58 | 4.92×10^-5^ |
| 19 | 57,466,663 | 57,477,570 | *ZNF772* | 0.81 | 5.20×10^-5^ | 0.81 | 4.40×10^-4^ | 0.91 | 3.16×10^-5^ |
| 22 | 42,509,968 | 42,519,802 | *RRP7A* | 0.43 | 4.02×10^-5^ | 0.45 | 3.89×10^-4^ | 0.28 | 2.91×10^-5^ |
| 21 | 36,069,941 | 36,073,166 | *CBR1* | 0.37 | 1.14×10^-4^ | 0.36 | 1.34×10^-3^ | 0.23 | 1.79×10^-4^ |
| 2 | 98,619,106 | 98,731,126 | *MGAT4A* | 2.81 | 8.58×10^-5^ | 2.91 | 4.25×10^-4^ | 2.53 | 1.21×10^-4^ |
| 1 | 145,911,350 | 145,918,837 | *PEX11B* | 0.66 | 5.54×10^-5^ | 0.68 | 2.53×10^-4^ | 0.63 | 5.97×10^-5^ |

^a^Model 1 further adjusted for childhood traumatic experience. ^b^Model 2 further adjusted for antidepressant usage. ^c^Model 3 further adjusted for use of antidepressants.
